# Stimulation of the Runx2 P1 promoter by collagen-derived dipeptide prolyl-hydroxyproline bound to Foxg1 and Foxo1 in osteoblasts

**DOI:** 10.1042/BSR20210304

**Published:** 2021-12-07

**Authors:** Kaho Nomura, Yoshifumi Kimira, Yoshihiro Osawa, Aya Kataoka-Matsushita, Koichi Takao, Yoshiaki Sugita, Jun Shimizu, Masahiro Wada, Hiroshi Mano

**Affiliations:** 1 Faculty of Pharmaceutical Sciences, Josai University, 1-1 Keyakidai, Sakado, Saitama 350-0295, Japan; 2 Nitta Gelatin Inc., 2-22 Futamata, Yao, Osaka 581-0024, Japan

**Keywords:** Foxg1, molecular interactions, osteogenesis, peptides, Runx2, transcription

## Abstract

Collagen-derived dipeptide prolyl-hydroxyproline (Pro-Hyp) directly binds to the forkhead box g1 (Foxg1) protein and causes it to undergo structural alteration. Pro-Hyp also promotes the production of a regulator of osteoblast differentiation, Runt-related transcription factor 2 (Runx2), through Foxg1, inducing osteoblast differentiation. In addition, Pro-Hyp disrupts the interaction between Foxg1 and Runx2, and Foxg1 appears to interact with Runx2 in the absence of Pro-Hyp. To elucidate the mechanism of Pro-Hyp that promotes osteoblast differentiation, we investigated whether Pro-Hyp regulates the Runx2 P1 promoter together with Foxg1. The present study revealed that Pro-Hyp is taken up by osteoblastic cells via the solute carrier family 15 member (Slc15a) 4. In the presence of Pro-Hyp, Runx2 is translocated from the nucleus to the cytoplasm and Foxg1 is translocated from the cytoplasm to the nucleus. We also found that Pro-Hyp promoted the interaction between Forkhead box o1 (Foxo1) and Runx2 and the dissociation of Foxg1 from Runx2. Moreover, we identified the Pro-Hyp response element in the *Runx2* distal P1 promoter at nt −375 to −316, including the Runx2 binding sites and Fox core sequence. In the presence of Pro-Hyp, Runx2 is dissociated from the Pro-Hyp response element in the *Runx2* distal P1 promoter. Subsequently, Foxg1 and Foxo1 activated the Runx2 promoter by binding to the Pro-Hyp response element. In summary, we delineated the mechanism by which Pro-Hyp stimulates the bone-related *Runx2* distal P1 promoter activity in osteoblastic cells through Foxg1, Foxo1, and Runx2.

## Introduction

Bones are composed of osteoblasts embedded in an extracellular matrix of organic compounds and inorganic compounds, mainly comprising type 1 collagen and hydroxyapatite, respectively. During bone formation, osteoblasts secrete type 1 collagen and osteocalcin, which play a central role [1]. During bone resorption, osteoclasts secrete both hydrochloric acid, which resorbs hydroxyapatite, and proteases, such as matrix metalloproteinase (MMP) and cathepsin K, which degrade collagen and other bone matrix proteins [2]. Bone formation and bone resorption are linked and regulated by coupling factors [3]. Balancing the two processes is essential for healthy bone formation.

Osteoblast differentiation is necessary for bone formation because osteoblasts secrete collagen and facilitate the deposition of hydroxyapatite in the bone matrix.

Bone turnover is evaluated by measuring the urinary N-telopeptide of type I collagen (NTX) and blood type I procollagen-N-propeptide (P1NP) [4]. In addition, prolyl hydroxyproline (Pro-Hyp), the main product of collagen degradation during bone resorption, has been detected in non-hydrolyzed serum and urine, and it could be a useful bone metabolism marker [5,6].

Interestingly, collagen hydrolysates are also used as functional foods [7]. When collagen hydrolysates are ingested, Pro-Hyp is detected at a high concentration in the blood [8,9]. Orally ingested [^14^C] Pro-Hyp is distributed in bone, cartilage, skin, and other tissues in rats [10]. Pro-Hyp has been reported to act on cell differentiation such as that of osteoblasts and fibroblasts [11–15]. Collagen-derived peptides are of exogenous as well as endogenous origins. We hypothesized that the Pro-Hyp was absorbed from the blood or produced by osteoclasts to up-regulate osteoblast differentiation as a coupling factor.

The extracellular Pro-Hyp has been reported to be absorbed by osteoblastic cells [16]. However, it is unclear how Pro-Hyp is incorporated into osteoblasts. In the small intestinal epithelial cells, Pro-Hyp is intracellularly transported via solute carrier family 15 member (Slc15a) 1 (Slc15a1) [17]. The *Slc15* family of electrogenic membrane transporters mediates the cellular uptake of dipeptides, tripeptides, and many peptidomimetics utilizing an inwardly directed proton gradient and negative membrane potential [18,19].

In humans, the SLC15 family has four members, PepT1, PepT2, PhT1, and PhT2, which are encoded by SLC15A1, SLC15A2, SLC15A4, and SLC15A3, respectively [20]. In mice, the Slc15 family has four members, Pept1, Pept2, Pht1, and Pht2, which are encoded by Slc15a1, Slc15a2, Slc15a4, and Slc15a3, respectively [20]. We assumed that Pro-Hyp is transported into osteoblastic cells via the Slc15 family.

Osteoblast differentiation is also stringently regulated by various transcription factors. However, the underlying mechanisms remain unclear. Transcription factor, Runt-related transcription factor 2 (Runx2) is a master regulator of bone development and an activator of osteoblast differentiation [21–24]. *Runx2* expression is regulated by the distal P1 promoter whose activity produces the N-terminal isoform type II Runx2, such as MASNS type and Runx2 p57/P1, and the proximal P2 promoter whose activity produces the N-terminal isoform type I Runx2, such as MRIPV type and Runx2 p56/P2. While type I Runx2 was first identified as a T-cell specific factor, type II Runx2 was initially revealed to be a bone-specific factor [25]. In osteoblasts, *Runx2* expression is tightly autoregulated through the negative feedback of its distal P1 promoter [26]. During osteoblast differentiation, the transcriptional control of *Runx2* is primarily mediated by the upstream P1 promoter [27,28]. *Runx2* distal P1 promoter becomes highly transcribed during osteoblast differentiation and is stimulated by several bones forming homeodomain and Hox proteins [29].

The family of forkhead transcription factors is also critically involved with Runx2 in bone development. For instance, Forkhead box o1 (Foxo1) is a regulator of bone formation [30–33]. During early osteoblast differentiation, amino acids 360–456 of Foxo1 directly interact with amino acids 242–258 of Runx2 to dissociate Runx2 from the osteocalcin promoter [34]. The forkhead transcription factor forkhead box c2 (Foxc2) also promotes osteoblast genesis [35].

In addition, we have demonstrated for the first time that forkhead box g1 (Foxg1) plays a vital role in *Runx2* mRNA expression by Pro-Hyp [36]. We found that when Pro-Hyp is directly bound to recombinant Foxg1, Foxg1 undergoes structural alteration in Foxg1. In addition, Pro-Hyp disrupts the interaction between recombinant Foxg1 and Runx2, and recombinant Foxg1 appears to interact with recombinant Runx2 in the absence of Pro-Hyp [37]. However, it is unclear how Foxg1–Runx2 heterodimers act on osteoblast differentiation. Foxg1 also plays a crucial role in the development of the cerebral cortex and inner ear [38]. Mouse Foxg1 contains a DNA-binding domain at amino acids 172–263 [39]. Fox transcription factors recognize the Fox core sequence 5′-(A/C)AA(C/T) A-3′ and bind to DNA to activate or suppress the expression of target genes [40].

We hypothesized that Pro-Hyp promotes *Runx2* distal P1 promoter activity through Foxo1, Foxg1, and Runx2. After oral ingestion of collagen hydrolysate (0.385 g/kg body weight), Pro-Hyp was detected in the blood at the *C*_max_ of 60.65 ± 5.74 μM [41]. A concentration of 0.1–1 mM of Pro-Hyp is known to induce osteoblast differentiation [16,42]. Therefore, Pro-Hyp was applied to experiments at 0.1–1 mM in this study.

In the present study, we elucidated the mechanism underlying the role of Pro-Hyp in osteoblast differentiation, identified the Pro-Hyp response element in the *Runx2* P1 promoter, and delineated how Pro-Hyp promotes Runx2 promoter activity through Foxg1, Foxo1, and Runx2. Our findings collectively indicate that the promoter activity of *Runx2*, which is the osteoblast master gene, is enhanced via the novel regulation of the interaction among Foxg1, Foxo1, and Runx2 by Pro-Hyp.

## Materials and methods

### Reagents

Pro-Hyp (Bachem) with a purity of 99% was dissolved in Minimum essential Eagle’s medium-α modification (αMEM, Gibco/Life Technologies) and stored at −20°C. Fetal bovine serum (FBS) (Sigma–Aldrich), Slc15a4 siRNA (Ambion®), and Control siRNA (Santa Cruz Biotechnology) were purchased.

Anti-Runx2 (cat. no. 8486), anti-Foxo1 (cat. no. 2880), secondary antibody (cat. no. 7076) (Cell Signaling Technology, Inc), and anti-Foxg1 (cat. no. ab18259) (Abcam) were procured for immunoprecipitation (IP) and Western blot. In addition, anti-Flag® M1 Agarose Affinity Gel (cat. no. A4596) (Merck) was obtained for IP.

Anti-Runx2 (cat. no. 12556), anti-Foxo1 (cat. no. 2880), and Anti-Alexa Fluor® 488 (cat. no.4412) (Cell Signaling Technology, Inc) and anti-Foxg1 (cat. no. ab18259) (Abcam) were obtained for immunofluorescence. Anti-Runx2 (cat. no. 8486) and anti-Foxo1 (cat. no. 2880) (Cell Signaling Technology, Inc), and Anti-Foxg1 (cat. no. ab18259) (Abcam) and normal rabbit IgG antibody (cat. no. 2729) (Cell Signaling Technology, Inc) were used for chromatin IP (ChIP).

### cDNA plasmids

*Foxg1*, *Foxo1*, and *Runx2* mouse cDNA were amplified using polymerase chain reaction (PCR) from single-strand cDNA obtained from a mouse brain and the mouse osteoblastic cell line MC3T3-E1. Full-length mouse *Foxg1*, *Foxo1*, and *Runx2* cDNA were subcloned into the expression vector pRK7-Myc to create vectors that express Myc-Foxg1, Myc-Foxo1, and Myc-Runx2 fusion proteins, respectively. Full-length mouse *Foxg1*, *Foxo1*, and *Runx2* cDNA were subcloned into the expression vector pRK7-Flag to create vectors that express Flag-Foxg1, Flag-Foxo1, and Flag-Runx2, respectively.

*Runx2* and *Foxo1* were cloned into a glutathione S-transferase (GST) fusion protein expression vector, as described previously [37]. In addition, fragments of Foxg1 were cloned into the same vector to express GST-Foxg1 mutants Foxg1A (amino acids 1–171), Foxg1B (amino acids 172–263), Foxg1C (amino acids 264–375), and Foxg1D (amino acids 376–481). All sequences were verified using automatic DNA sequencing. In addition, the production of Foxo1 was confirmed using Western blot analysis.

### RNA isolation and RT-PCR

Total RNA was isolated from MC3T3-E1 cells using an RNeasy Mini Kit (Qiagen). One microgram of total RNA was reverse transcribed using the PrimeScript™ RT-PCR Kit (Takara) with appropriate primers (Supplementary Data S1). The reverse transcription-polymerase chain reaction (RT-PCR) was conducted using a GeneAmp® PCR System 9700 (Applied Biosystems) with 50 cycles of 94°C for 30 s, 60°C for 30 s, and 72°C for 3 min. The PCR products were resolved on 1% agarose gels.

### Transfection of siRNA into MC3T3-E1

MC3T3-E1 cells were plated in 96-well plates or 8-well chamber slides in αMEM with 10% FBS, transiently transfected with 10 nM of *Slc15a4* or Control siRNA using Lipofectamine Reagent (Life Technologies), and cultured in the presence or absence of 0.1 mM Pro-Hyp.

### Preparation of fluorescein isothiocyanate-labeled Pro-Hyp

Twenty milligrams of Pro-Hyp was dissolved in 1 ml of 0.1 M NaHCO_3_ and mixed with 40 mg of fluorescein isothiocyanate (FITC) in 1 ml of N,N-dimethylformamide. The mixture was wrapped with aluminum foil and kept at room temperature for 2 h. The reaction was monitored using thin-layer chromatography (TLC). The FITC-labeled Pro-Hyp was purified using a preparative TLC plate with chloroform:methanol:acetic acid (3:1:0.1) as the solvent, resulting in a 79.9% yield at 42.1 mg. The structure of FITC-labeled Pro-Hyp was confirmed with ^1^H- and ^13^C-nuclear magnetic resonance spectroscopy.

### Incorporation of FITC-labeled Pro-Hyp into MC3T3-E1

The MC3T3-E1 cells were treated with 10 μM of FITC-labeled Pro-Hyp or uranine (Control), with 50 mM of glycine, 50 mM of histidine, or sterile water (Control) for 48 h. The cells were rinsed three times with phosphate-buffered saline (PBS), fixed with 4% paraformaldehyde, nuclear stained with propidium iodide (PI) at 1:1000, and observed using fluorescence microscopy (KEYENCE, Osaka, Japan).

### Immunofluorescence

The MC3T3-E1 cells were treated with a medium containing 0.1 mM Pro-Hyp for 48 h. After fixing with 4% paraformaldehyde, the cells were incubated with primary antibodies of Foxg1 at 1:100, Foxo1 at 1:100, or Runx2 at 1:100 at 4°C overnight. The next day, the samples were incubated with anti-Alexa Fluor® 488 secondary antibodies at 1:1000 at 4°C overnight. The cells were then nuclear stained with PI at 1:1000 and observed using fluorescence microscopy (KEYENCE, Osaka, Japan).

### Cell culture

Mouse calvaria osteoblastic MC3T3-E1 cells were cultured in a growth medium comprising αMEM supplemented with 10% FBS and 100 U/ml penicillin. HEK293T cells were cultured in Dulbecco’s modified Eagle’s medium (Gibco, Carlsbad, CA, U.S.A.) supplemented with 10% FBS and 100 U/ml penicillin. The cell cultures were maintained at 37°C in a humidified chamber at 5% CO_2_.

### IP

We examined the effect of Pro-Hyp on the interaction among Foxg1, Foxo1, and Runx2. HEK293 cells were co-transfected with Myc-Runx2 and Flag-Foxg1 or Flag-Foxo1 or Flag-Runx2 and Myc-Foxg1. After 48 h, cell extracts were collected for IP assay using an anti-Flag M2 affinity gel. The specificity of the IP assay was confirmed using Western blot with the anti-Runx2 antibody. Experiments were repeated three times with similar results.

We examined whether Pro-Hyp influenced the interaction between Foxo1 and Runx2. We determined whether Foxg1 interacted with Runx2 by performing IP assays. We also examined whether Pro-Hyp influences Foxg1 and Runx2 interaction by performing IP assays with HEK293T cells; and we found that Pro-Hyp dissociated the interaction between Foxg1 and Runx2 in the presence of nuclear proteins.

### GST pull-down

Purified GST fusion proteins were used for IP with specific antibodies, as previously described [19]. Truncated mutants of GST-Foxg1 corresponding to different regions of Foxg1 and GST-Foxo1 were purified using Glutathione Sepharose 4B beads (GE Healthcare). Each purified truncated mutants of GST-Foxg1 or GST-Foxo1 recombinant proteins were incubated with recombinant Runx2 on ice for 3 h. Each truncated mutant of GST-Foxg1 or GST-Foxo1 and Runx2 heterodimer was precipitated with Glutathione Sepharose 4B beads. The beads were then washed with PBS before protein elution. The presence of Runx2 in the precipitates was detected using Western blot and the rabbit anti-Runx2 antibody.

### Ligand binding assay

Pro-Hyp-Gly pentamer (Pro-Hyp-Gly)_5_ was linked to magnetic beads (TAMAGAWA Seiki, Nagano, Japan) by its N-terminus. The (Pro-Hyp-Gly)_5_-linked magnetic beads were equilibrated with a binding buffer comprising 150 mM KCl, 10 mM HEPES (pH = 7.8), 1 mM EDTA, 10% Glycerol, 1 mM DTT, and Protease inhibitor tablets, and then incubated with 2 mg of MC3T3-E1 cell extraction at 4°C overnight with occasional agitation. Free Pro-Hyp was added at a concentration of 0.1 or 10 mM to perform the competition assay and confirm the binding specificity of a protein to Pro-Hyp. After washing the beads with the binding buffer three times, the bound proteins were eluted with sodium dodecyl sulfate polyacrylamide gel electrophoresis (SDS/PAGE) sample buffer and subjected to SDS/PAGE and Western blot. Experiments were repeated three times with similar results.

We considered that Pro-Hyp-linked magnetic beads could not detect Foxo1 bound to Pro-Hyp, as the molecular size of Pro-Hyp is very small. Thus, to facilitate the sensitivity detection of Pro-Hyp-bound Foxo1, we used (Pro-Hyp-Gly)_5_-linked magnetic beads. In addition, considering that repeated binding of Pro-Hyp causes steric hindrance, Gly, which has a low molecule weight and neutral amino acid, was inserted between Pro-Hyp and Pro-Hyp.

### Construction of luciferase reporter plasmids

Each region of the mouse *Runx2* distal P1 promoter was amplified using PCR with genomic DNA extracted from the mouse osteoblastic cell line MC3T3-E1. Oligonucleotides were annealed in 50-µl reactions containing 10 µM of the upper and lower strands of the oligonucleotides (Table 1), sodium chloride-Tris-EDTA buffer containing TE, 5 M NaCl, and Milli-Q water. The solution was heated to 99°C for 10 min, to 90°C for 5 min, and then cooled to 30°C at 5°C per min. These oligonucleotides were then subcloned into XhoI-digested pGL3-control plasmid, which contained an SV40 promoter upstream of a luciferase gene and an SV40 enhancer of a luciferase gene. The sequence of the subcloned vectors was confirmed via sequencing.

### Luciferase reporter assay

The MC3T3-E1 cells were cultured in 96-well plates. The cells were then transfected with 100 ng of the pGL3-control plasmid containing the *Runx2* distal P1 promoter with wildtype (WT) or mutated Runx2-binding sites and 1 ng of pNL (Promega) using Lipofectamine 2000 following the manufacturer’s instructions (Thermo Fisher Scientific). The cells were incubated for 48 h before the transfection reagents were removed, and Pro-Hyp was added for analysis.

Luciferase assays were performed using the Dual-Glo Luciferase Assay System (Promega). After the Pro-Hyp treatments were completed, 80 µl of Dual-Glo reagent was added to each well, and then samples were incubated at room temperature for 10 min. The firefly luciferase luminescence was recorded using a Glo-Max Discover Microplate Reader with an integration time of 1 s.

Eighty microliters of Dual-Glo ‘Stop & Glo’ reagent was then added to each well and incubated at room temperature for 5 min before recording the NanoLuc luminescence. The firefly:NanoLuc luminescence ratio was calculated to determine the activity of different *Runx2* distal P1 promoter sites.

### ChIP

We performed chromatin immunoprecipitation/quantitative polymerase chain reaction (ChIP/qPCR) analysis using MC3T3-E1 cells with and without Pro-Hyp treatment for 48 h to determine whether osteoblast differentiation transcription factors associated with the endogenous *Runx2* distal P1 promoter *in vivo*.

The ChIP experiment was performed to detect the effects of Pro-Hyp on the promoter activity of Runx2 in the osteoblastic cells.

ChIP experiments were performed on MC3T3-E1 cells using the ChIP Assay Kit (Millipore), an anti-Runx2 antibody, anti-Foxo1 antibody, anti-Foxg1 antibody, and normal rabbit IgG following the manufacturer’s instructions.

The cells were cross-linked with 1% formaldehyde for 15 min at 37°C, washed, collected in cold PBS, lysed in SDS lysis buffer containing protease inhibitors, and sonicated to produce 200–1000 bp DNA fragments. The sonicated samples were incubated with 2 µg of rabbit anti-Runx2 antibody, rabbit anti-Foxo1 antibody, rabbit anti-Foxg1 antibody, or normal rabbit IgG overnight at 4°C. The antibody–protein–DNA complex was enriched by mixing with ChIP-grade protein A/G-agarose beads for 1 h at 4°C. The bound DNA was purified following the instructions provided with the ChIP Assay Kit, amplified, and quantified using qPCR with specific primers (Table 2).

### Statistical analyses

The error bars present the standard deviation (Figures 3D, Figure 4C, Figure 5B-F, Figure 6B-E). The Student’s paired two-tailed *t* test was used to determine the significance of the differences between the experimental groups; *P*-values <0.05 were considered significant (Figure 3D, Figure 4C, Figure 6B-E). After performing a one-way analysis of variance, the Tukey’s post hoc test was used to compare the differences between the means at the 5% probability level (*P*<0.05) (Figure 5B–F).

## Results

### Pro-Hyp is incorporated into osteoblastic cells via Slc15a4

**Figure 1:**
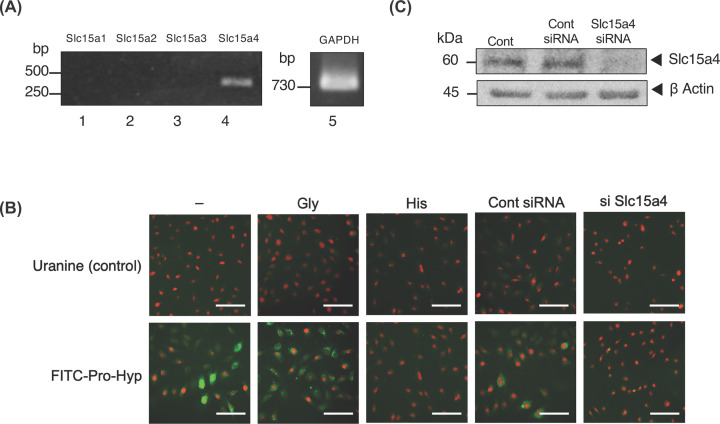
Pro-Hyp is incorporated into osteoblastic cells via Slc15a4 (**A**) The expression of the *Slc15a* family of genes, which encodes peptide transporters, in osteoblastic cells is assayed using RT-PCR. (**B**) The untransfected MC3T3-E1 cells treated with PBS, glycine, or histidine, MC3T3-E1 cells transfected with Control siRNA, and MC3T3-E1 with knocked down *Slc15a4* were treated with uranine (upper panel) (green) or FITC-labeled-Pro-Hyp (lower panel) (green) for 48 h. After incubation, the nuclei were stained with PI (red). The images were captured using a KEYENCE fluorescent microscope. Scale bar, 100 µm. The data are representative of three independent experiments. (**C**) *Slc15a4* was knocked down in MC3T3-E1 cells. Cell lysates were collected and expression levels of Slc15a4 was analyzed via Western blotting.

We sought to identify the transporter used to incorporate Pro-Hyp into osteoblastic cells. First, we examined the expression of different *Slc15* genes in osteoblasts. Only *Slc15a4* mRNA (Figure 1A, lane 4), but not *Slc15a1–3* mRNA (Figure 1A, lanes 1–3), was observed in the MC3T3-E1 cells.

We then investigated whether Pro-Hyp was incorporated into MC3T3-E1 cells through Slc15a4. The MC3T3-E1 cells were treated with uranine (Control) or FITC-labeled Pro-Hyp for 48 h. Green fluorescence was observed in MC3T3-E1 cells treated with FITC-Pro-Hyp but not in uranine-treated MC3T3-E1 cells (Figure 1B).

Histidine is an Slc15a4 inhibitor [43]. Uranine or FITC-labeled Pro-Hyp-treated MC3T3-E1 cells were incubated with glycine (control) or histidine for 48 h. Subcellular green fluorescence decreased in cells treated with FITC-Pro-Hyp and histidine but when treated with the control amino acid glycine and FITC-Pro-Hyp no changed intensity of subcellular green fluorescence (Figure 1B).

We knocked down the expression of endogenous Slc15a4 using siRNA. The knocked down of *Slc15a4* in MC3T3-E1 cells was confirmed with Western blot, which indicated the reduced expression of *Slc15a4* (Figure 1C). When the *Slc15a4*-knocked down MC3T3-E1 cells were treated with FITC-Pro-Hyp, subcellular green fluorescence was not observed. When the control-knocked down (Control siRNA) MC3T3-E1 cells were treated with FITC-Pro-Hyp, subcellular green fluorescence was observed (Figure 1B). These results suggest that Pro-Hyp is incorporated into MC3T3-E1 cells via Slc15a4 and is localized to the cytoplasm and nucleus.

### Pro-Hyp regulates the subcellular localization of Foxg1, Foxo1, and Runx2 in osteoblastic cells

We next investigated the subcellular distribution of Foxg1, Foxo1, and Runx2 in the presence or absence of Pro-Hyp with immunofluorescence.

**Figure 2:**
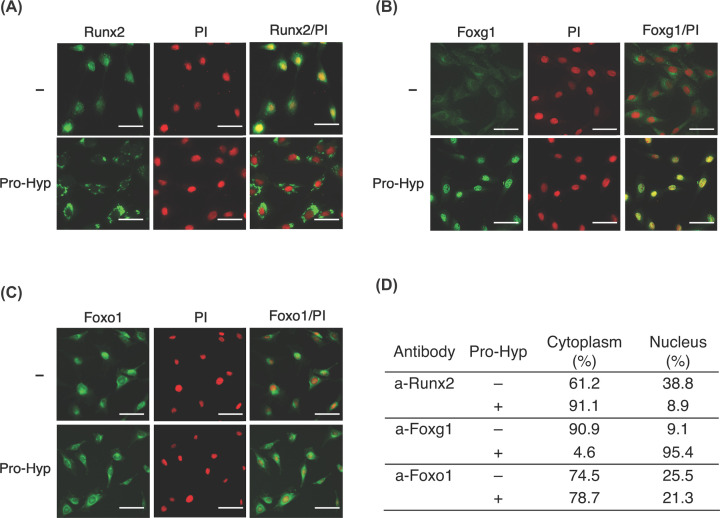
Pro-Hyp promotes the translocation of Foxg1, Foxo1, and Runx2 in osteoblast cells Immunofluorescence staining of Runx2 (**A**), Foxg1 (**B**), and Foxo1 (**C**) in MC3T3-E1 cells treated with Pro-Hyp or the control with anti-Foxg1 (green), anti-Foxo1 (green), and anti-Runx2 (green) antibodies. The cells were also nuclear stained with PI (red). Representative images were quantified (**D**) to calculate the fluorescence intensity of the transcription factor localized to the nuclei or the cytoplasm. Scale bar, 50 µm. The data were representative of three independent experiments.

In the absence of Pro-Hyp, a portion of Runx2 was detected in the nucleus. In the presence of Pro-Hyp, Runx2 was mainly detected in the cytoplasm (Figure 2A). In the absence of Pro-Hyp, Foxg1 was primarily detected in the cytoplasm. Surprisingly, in the presence of Pro-Hyp, Foxg1 could be detected in the nucleus (Figure 2B). Pro-Hyp did not affect the subcellular distribution of Foxo1 (Figure 2C).

**Figure 3:**
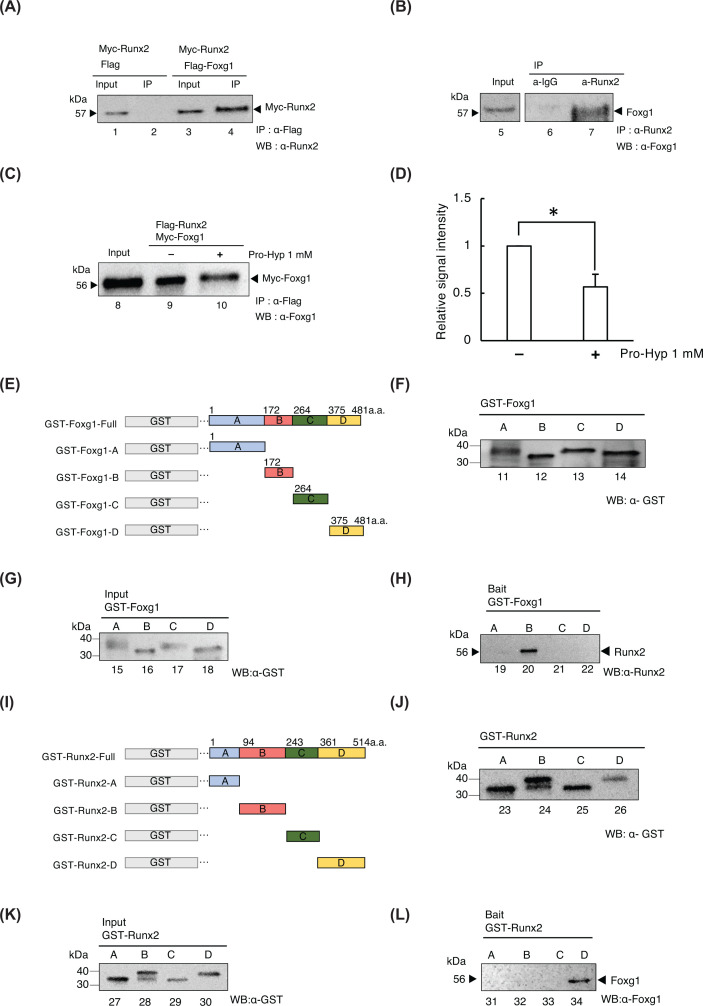
Pro-Hyp inhibits the interaction between Foxg1 and Runx2 (**A**) IP assays using the extracts from HEK293T cells overexpressing *Flag-Foxg1* and *Myc-Runx2*. (**B**) IP assay using cell extract from MC3T3-E1. Extracts were immunoprecipitated with control rabbit IgG (lane 6), anti-Flag antibody (lanes 2, 4, 9, and 10), or anti Runx2 antibody (lane 7) followed by Western blot analysis using respective antibodies. (**C**) IP assays using the extracts from HEK293T cells overexpressing *Flag-Runx2* and *Myc-Foxg1*. Pro-Hyp was added at a concentration of 1 mM (lane 10). (**D**) The amount of immunoprecipitated Myc-Foxg1 was evaluated using ImageJ software. Data are presented as means ± SD (*n*=3). *P<0.05. (**E**) Schematic showing the regions of Foxg1 and the truncated mutants. Foxg1-A: amino acids 1–171; Foxg1-B: amino acids 172–263; Foxg1-C: amino acids 264–374; and Foxg1-D: amino acids 375–481. (**F**) GST tag was added to the constructs encoding the mutant Foxg1 proteins to produce various GST-Foxg1 truncated mutants, including GST-Foxg1-A 43 kDa (lane 11), GST-Foxg1-B 36 kDa (lane 12), GST-Foxg1-C 37 kDa (lane 13), and GST-Foxg1-D 36 kDa (lane 14). (**H**) GST pull-down assay. The Foxg1 truncated mutants with Runx2 were mixed (lanes 19–22). They were then analyzed by Western blot analysis using the anti-Runx2 antibody. Foxg1 truncated mutant protein extracts were used as the input (lanes 15–28) (**G**). (**I**) Schematic showing the region structure of Runx2 and its truncated mutants. Runx2-A: amino acids 1–93; Runx2-B: amino acids 94–243; Runx2-C: amino acids 243–360; and Runx2-D: amino acids 361–514. (**J**) GST tag was added to the Runx2 mutants via cloning. The various GST-Runx2 truncated mutants included GST-Runx2-A 35 kDa (lane 27), GST-Runx2-B 41.6 kDa (lane 28), GST-Runx2-C 37.6 kDa (lane 29), and GST-Runx2-D 41.6 kDa (lane 30), as visualized by Western blot for GST. (**L**) GST pull-down assay. The Runx2 truncated mutants with Foxg1 were mixed (lanes 31–34) and then analyzed using Western blot with the anti-Foxg1 antibody. (**K**) The Runx2 truncated mutant protein extracts were used as the input (lanes 27–30).

**Figure 4:**
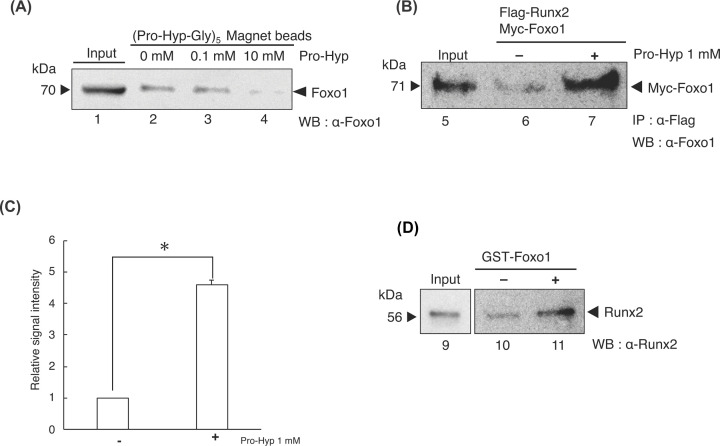
Pro-Hyp promotes the interaction between Foxo1 and Runx2 (**A**) Ligand binging assay. MC3T3-E1 cell extracts were mixed with (Pro-Hyp-Gly)_5_-magnetic beads. A competition inhibition assay was then performed by adding an excess amount of Pro-Hyp at a concentration of 0.1 mM (lane 3) and 10 mM (lane 4), followed by the Western blot analysis of Foxo1. Pro-Hyp was not added to lane 2. An amount of 0.1 μg of cell extracts was used as input (lane 1). (**B**) IP assays using extracts from the HEK293T cells overexpressing *Flag-Runx2* and *Myc-Foxo1*. Pro-Hyp was added at a concentration of 1 mM (lane 7). Extracts were immunoprecipitated with the Flag antibody, followed by Western blot analysis using the Foxo1 antibody. (**C**) The amount of immunoprecipitated Myc-Foxo1 was evaluated using ImageJ software. Data are presented as means ± SD (*n*=3). *P<0.05. (**D**) GST-Foxo1 with Runx2 were mixed (lanes 10 and 11). Pro-Hyp was added at a concentration of 1 mM (lane 11). Foxo1 binding to (Pro-Hyp-Gly)_5_-magnetic beads in a Pro-Hyp-specific manner.

**Figure 5:**
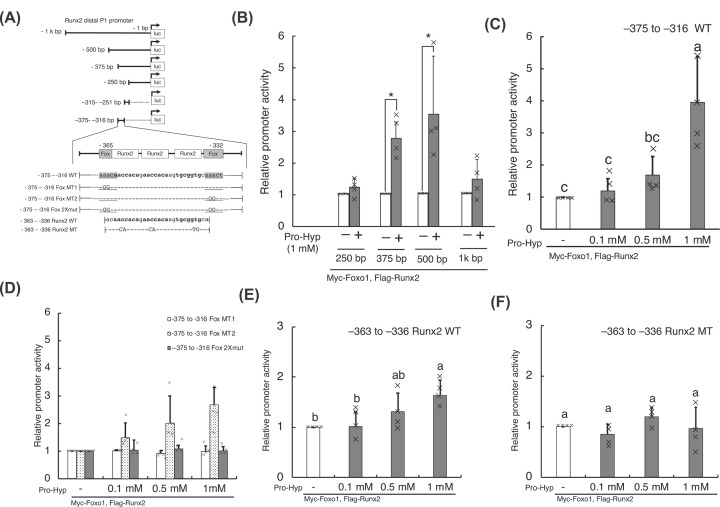
Identification of Pro-Hyp response element in the proximal region of the Runx2 distal P1 promoter via Foxg1 and Foxo1 Schematic illustration of the luciferase expression vectors containing different regions of the *Runx2* distal P1 promoter (**A**). Boldface nucleotides represent the wildtype Runx2-binding sequence (WT). The solid underlined region represents the wildtype Fox consensus sequence (WT). The uppercase nucleotides represent the mutations (MT) introduced into the pGL3 control vector. The MC3T3-E1 cells were transfected with 0.1 µg of pGL3-*Runx2* distal P1 promoter DNA and 1 ng pNL DNA. After 48 h, the cells were treated with Pro-Hyp for 48 h, as indicated (**B–F**). Luciferase activity was measured and normalized to the activity of NanoLuc luciferase. Luciferase assay was used to identify the Pro-Hyp response element in the proximal region of the *Runx2* distal P1 promoter (B). The effect of the mutations of the Fox core sequence in the proximal region of the *Runx2* distal P1 promoter on the induction of *Runx2* distal P1 promoter activity by Pro-Hyp was tested (C,D). The effect of the mutations in the proximal region of the *Runx2* distal P1 promoter on the promotion of the *Runx2* distal P1 promoter activity by Pro-Hyp was tested (E,F). Significance was calculated using the Student’s *t* test (B) and Tukey’s post hoc test (C–F); the mean error bars represent the standard deviation. For clarity, not all the significant differences are indicated. Each data point is the mean ± S.D. of four independent assays. *P<0.05.

**Figure 6:**
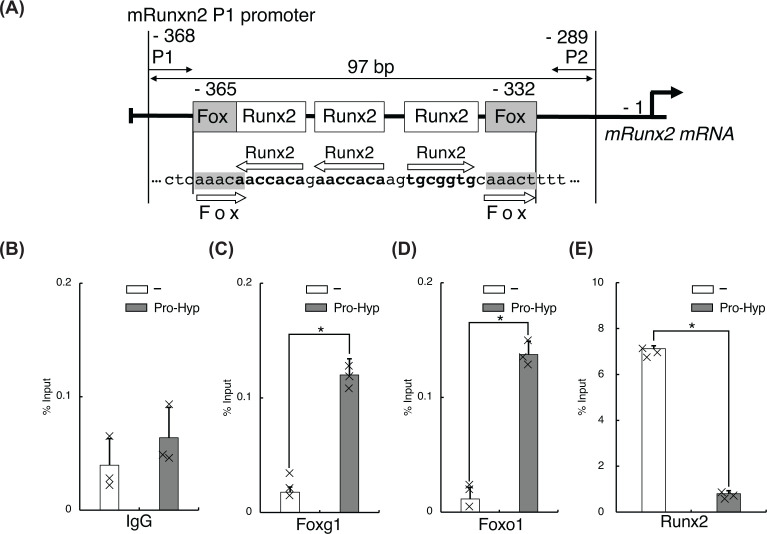
A complex of Pro-Hyp, Foxg1, and Foxo1 binding to the proximal region of Runx2 distal P1 promoter Verification of the Pro-Hyp response element in the Runx2 distal P1 promoter, including the Runx2-binding site. (**A**) A schematic representation of the relevant regions of the *Runx2* distal P1 promoter. P1, P2 indicate PCR primers used to analyze ChIP/qPCR. The positions of these primers and the size of the amplified fragments are indicated at the top of the figure. (**B–E**) ChIP/qPCR analysis of the *Runx2* distal P1 promoter in MC3T3-E1 cells. Cells were incubated with and without 1 mM Pro-Hyp for the 48 h, followed by ChIP/qPCR analysis using a control Rabbit IgG antibody (B), Foxo1 antibody (C), Foxo1 antibody (D), or Runx2 antibody (E). Experiments were repeated three times with similar results. Data are presented as means ± standard deviation (*n*=3), *P<0.05. Significance was calculated using the Student’s *t* test.

We then investigated whether Pro-Hyp modulated transcription factor localization. We measured the area of staining of the transcription factors in the nucleus and cytoplasm and calculated the percentage of nuclear and cytoplasmic localization of the transcription factors (Figure 2D). Pro-Hyp was found to moderate Runx2’s translocation from the nucleus to the cytoplasm. In contrast, Foxg1 translocates from the cytoplasm to the nucleus in the presence of Pro-Hyp.

### Pro-Hyp inhibits physical interaction between Foxg1 and Runx2

We previously demonstrated that Pro-Hyp bound to the DNA-binding domain of Foxg1, disrupts the interaction between Foxg1 and Runx2 in the absence of nuclear proteins [37]. Here, we examined whether Pro-Hyp dissociated the interaction between Foxg1 and Runx2 in the presence of cytosolic proteins or nuclear proteins. Myc-Runx2 was immunoprecipitated from HEK293T cells that were exogenously expressed Myc-Runx2 and Flag-Foxg1 (Figure 3A, lane 4). There was no IP of Myc-Runx2 from HEK293T cells exogenously expressing Myc-Runx2 and Flag (Figure 3A, lane 2).

Next, we performed IP assays using extracts from MC3T3-E1 osteoblastic cells. Endogenous Foxg1 protein was present in an IP with an anti-Runx2 antibody (Figure 3B, lane 7) but not in IP with an anti-IgG antibody (Figure 3B, lane 6). Myc-Foxg1 was immunoprecipitated in greater quantity in the absence of Pro-Hyp than in the absence of Pro-Hyp (Figure 3C,D), suggesting that Pro-Hyp inhibited the interaction between Foxg1 and Runx2.

Because the respective binding regions of Foxg1 and Runx2 have not been investigated yet, we detected to examine these binding regions using GST pull-down assays. Various recombinant GST-Foxg1 deletion mutants, such as Foxg1-A (amino acids 1–171), Foxg1-B, (amino acids 172–263), Foxg1-C (amino acids 264–375), and Foxg1-D (amino acids 376–481), were generated in *Escherichia coli* (Figures 3E,F). We observed that GST-Foxg1-B, a DNA-binding region was bound to Runx2 with high affinity (Figure 3H, lane 20). Conversely, we observed no binding between Runx2 and GST-Foxg1-A, C, or D (Figure 3H, lanes 19, 21, and 22).

Similarly, various recombinant GST–Runx2 deletion mutants, including Runx2-A (amino acid 1–93), Runx2-B (amino acid 94–243), Runx2-C (amino acid 243–360), and Runx2-D (amino acid 361–514), were generated (Figure 3I,J). We observed that GST-Runx2-D, with a nuclear matrix targeting signal (NMTS), was bound to Foxg1 with high affinity (Figure 3L, lane 34). Conversely, no binding was observed between Foxg1 and GST-Runx2-A, B, or C (Figure 3H, lanes 31–33). These results demonstrate that Pro-Hyp dissociates the interaction between Foxg1 and Runx2. In addition, this interaction requires the DNA-binding domain of Foxg1 and the C-terminal region of Runx2.

### Pro-Hyp binds to Foxo1 and promotes interaction between Foxo1 and Runx2

Next, we tested whether Pro-Hyp bound to Foxo1. We prepared the recombinant Foxo1 and (Pro-Hyp-Gly)_5_-linked magnetic beads or the control magnetic beads for the ligand-binding assay. The binding between Foxo1 and (Pro-Hyp-Gly)_5_-linked magnetic beads were affected by Pro-Hyp in a concentration-dependent manner (Figure 4A, lanes 3 and 4). Foxo1 and (Pro-Hyp-Gly)_5_-linked magnetic beads completely dissociated at a Pro-Hyp concentration of 10 mM (Figure 4A, lane 4). Foxo1 did not bind to the control magnetic beads (Supplementary Data S2). These data suggest that Pro-Hyp binds to Foxo1.

In addition, we determined whether Pro-Hyp modulated the interaction between Foxo1 and Runx2 using an IP assay. In the absence of Pro-Hyp, Flag-Runx2 interacted weakly with Myc-Foxo1 (Figure 4B, lane 6). Surprisingly, the presence of Pro-Hyp significantly promoted the interaction between Myc-Foxo1 and Flag-Runx2 (Figure 4B lane 7, C). We also determined whether Pro-Hyp promoted the interaction between Foxo1 and Runx2 in the absence of other proteins. We conducted a GST-pull down assay using purified GST-Foxo1 and recombinant Runx2. Similarly, we found that GST-Foxo1 weakly bound to Runx2 (Figure 4D, lane 10). The presence of Pro-Hyp promoted the interaction between GST-Foxo1 and Runx2 (Figure 4D, lane 11). These data suggest that Pro-Hyp binds to Foxo1 and promotes the interaction between Foxo1 and Runx2.

### Identification of Pro-Hyp response element in the Runx2 distal P1 promoter

We sought to define the molecular mechanism underlying the promotion of *Runx2* expression by Pro-Hyp. We determined the Pro-Hyp response element in the *Runx2* distal promoter using a luciferase reporter assay. The luciferase reporter plasmid used is shown in Figure 5A. Vectors pRk7-Myc-*Foxo1* and pRK7-Flag-*Runx2*, encoding Myc-Foxo1 and Flag-Runx2, respectively, were co-transfected into MC3T3-E1 cells to determine the effects of Foxg1, Foxo1, Runx2 on the *Runx2* distal P1 promoter.

The level of firefly luciferase increased by approximately 2.79- and 3.57-fold in the cells transfected with the −375- and −500-bp of the *Runx2* P1 promoter, respectively, in the presence of 1 mM Pro-Hyp compared with those in the absence of Pro-Hyp (*P*<0.05, Figure 5B). We predicted that the Pro-Hyp response element was located in nt −375 to −250 in the *Runx2* distal P1 promoter. We performed luciferase reporter assay in the previous conditions but with the nt −375 to −316 or nt −315 to −251 of the *Runx2* P1 promoter. The response of the luciferase reporter to nt −375 to −316 depended on Pro-Hyp concentration (Figure 5C). In contrast, its the response to nt −315 to −251 was not affected by the treatment of 1 mM Pro-Hyp (Supplementary Data S3).

The *Runx2* distal P1 promoter nt −375 to −316 encompasses *Runx2* binding sites nt −362 to −356, nt −354 to −349, and nt −344 to −332 and Fox consensus sequences nt −365 to −361 and nt −337 to −332 (Figure 5A) [29,40]. We examined whether the Fox core sequences (‘Fox core sequence 1 and 2’) were required to induce *Runx2* promoter activity by Pro-Hyp. We used nt −375 to −316 of the *Runx2* distal P1 promoter containing a mutation in the Fox core sequence 1 and 2 (Figure 5A) for luciferase assay. Luciferase activity was detected in the nt −375 to −316 Fox mutation (MT) 2, but not in −375 to −316 Fox MT1 containing a mutation in the Fox core sequence 1 (Figure 5D).

We also determined the impact of the Runx2 binding site on the *Runx2* distal P1 promoter activity.

The nt −363 to −336 WT was inserted into the firefly luciferase reporter plasmid pGL3-control. The nt −363 to −336 with the three-point mutation (MT) in the Runx2-binding site (‘Runx2 binding site 1, 2, and 3’) was inserted into the pGL3-cont to disrupt the consensus Runx2-binding sequence (Figure 5A). When nt −363 to −336 WT was transfected into the osteoblastic cells, the expression of firefly luciferase increased by approximately 1.6-fold in the 1 mM Pro-Hyp treatment group compared with the group with no Pro-Hyp treatment (*P*<0.05, Figure 5E). Notably, no response to Pro-Hyp was observed in cells transfected with the nt −363 to −336 MT (Figure 5F). Therefore, we determined the Pro-Hyp response element in the *Runx2* distal P1 promoter at nt −365 to −332.

### Pro-Hyp help Foxg1 and Foxo1 bind to the Pro-Hyp response element in the Runx2 distal P1 promoter in osteoblastic cells

Runx2 interacts specifically with a chromatin fragment of the *Runx2* distal P1 promoter that contains the Runx2-binding site (nt −368 to −289) (Figure 6A). The binding of Foxg1 and Foxo1 to the Runx2 distal P1 promoter was examined using ChIP/qPCR and specifically designed primers (Figure 6A and Table 2). In the absence of Pro-Hyp, theinteraction of Foxg1 and Foxo1 with the *Runx2* distal P1 promoter was not observed. In the presence of Pro-Hyp, Foxg1, and Foxo1 were significantly bound to the *Runx2* distal P1 promoter (Figure 6C, D). In contrast, Runx2 protein was noticeably dissociated from the *Runx2* distal promoter with Pro-Hyp treatment (Figure 6E). These data suggest that Pro-Hyp dissociates Runx2 from the *Runx2* P1 distal promoter. Conversely, Pro-Hyp promotes the binding of Foxg1 and Foxo1 to the *Runx2* P1 distal promoter.

## Discussion

In the present study, we demonstrated the mechanism underlying the role of Pro-Hyp during osteoblast differentiation. First, we demonstrated that Pro-Hyp was incorporated into osteoblastic cells through Slc15a4. In the small intestinal epithelial cells, Pro-Hyp is transported into intracellular via Slc15a1 [17]. There have been no reports stating that other members of the Slc15a family participate in the uptake of Pro-Hyp. Accordingly, the specific transporter used to incorporate Pro-Hyp into cells depends on the target cell.

Second, we demonstrated that Pro-Hyp promotes Foxg1’s translocation from the cytoplasm into the nucleus; conversely, Runx2 is translocated from the nucleus into the cytoplasm in the presence of Pro-Hyp.

We previously reported that Pro-Hyp induced the structural alteration of the C-terminus of Foxg1 [41]. This structural alteration of Foxg1 might affect its intracellular localization. Nuclear Fox proteins act as transcriptional regulators, whereas cytoplasmic Fox proteins are inactive and often subjected to proteasomal degradation [44]. It has been reported that the subcellular localization of Runx2 is regulated by phosphorylation. Collagen-derived dipeptide hydroxyprolyl glycine (Hyp-Gly) activates phosphorylation signals [45,46]. Therefore, further studies are necessary to elucidate the effect of Pro-Hyp on phosphorylation signaling [47].

Recently, it was reported that small compounds regulate transcription by modulating the protein interaction such as Foxo1 and Runx2 [48,49]. Therefore, we believe that Pro-Hyp possibly regulates *Runx2* transcription by modulating protein interaction in the osteoblast.

We elucidated that Pro-Hyp modulated protein interaction, such as preventing the interaction between Foxg1 and Runx2 and promoting the interaction between Foxo1 and Runx2 in the absence of cytosolic proteins or nuclear proteins. We demonstrated for the first time that the interaction regions of Foxg1 and Runx2 are the Foxg1 DNA-binding domain and Runx2 C-terminal region, respectively. The C-terminal segment of Runx factors, which contains NMTS, regulates intranuclear mobility by increasing the association of Runx factors at the subnuclear foci in living cells [50]. This study reveals that Pro-Hyp abolishes the interaction between Runx2 and Foxg1 and promoted to export of dissociated Runx2 from nuclear of MC3T3-E1 to cyteplasm. This finding is in agreement with the localization of Runx2 when Pro-Hyp is incorporated into osteoblasts. Drissi et al*.* reported that Runx2 down-regulated the *Runx2* distal P1 promoter activity [26]. During early osteoblast differentiation, Foxo1 (amino acids 360 to 456) directly interacts with Runx2 (amino acids 242–258), resulting in the dissociation of Runx2 from the osteocalcin promoter [34]. Promotion of the interaction between Foxo1 and Runx2 by Pro-Hyp may cause Runx2 to dissociate from the *Runx2* promoter.

Finally, we determined the Pro-Hyp response element in the *Runx2* P1 promoter and searched for the transcription factor that bound to it. We identified the nt −365 to −332 in the *Runx2* P1 distal promoter as a Pro-Hyp response element using a luciferase reporter assay. We previously clarified that Pro-Hyp promoted osteoblast differentiation by enhancing *Runx2* expression by approximately 1.3-fold. In addition, Pro-Hyp promoted the expression of *Foxg1* by approximately three-fold. However, Pro-Hyp did not affect *Foxo1* expression [36]. We sought to clarify the effect of Foxg1, Foxo1, Runx2 on the *Runx2* distal promoter via Pro-Hyp in this study. *Myc-Foxo1* and *Flag-Runx2* were co-transfected into osteoblasts using the luciferase reporter assay. When *Myc-Foxg1*, *Myc-Foxo1*, and *Flag-Runx2* were also co-transfected into osteoblastic cells, Pro-Hyp promoted the activity of the *Runx2* distal P1 promoter (Supplementary Data S4). When *Foxg1* and *Foxo1* were transfected into HEK293 cells without *Runx2*, the induction of *Runx2* promoter activity by Pro-Hyp was not observed. Therefore, we hypothesized that the induction of *Runx2* P1 distal promoter activity by Pro-Hyp required the elimination of Runx2 autoregulation.

The Pro-Hyp response elements in the *Runx2* distal P1 promoter include Runx2 binding sites nt −362 to −356, nt −354 to −349, and nt −344 to −332 and Fox consensus sequences nt −365 to −361 and nt −337 to −332 [29,40]. Fox core sequence 1 corresponds to the Fox consensus sequence. Fox core sequence 2 and the Fox consensus sequence differ by 1 base. Pro-Hyp response was not detected on the *Runx2* promoter with mutations in the Fox core (MT1) and the Runx2 binding sites. These results suggest that the Runx2 binding site and Fox core sequence 1 play a crucial role in the response of Pro-Hyp. These results suggest that Pro-Hyp promotes Runx2 transcription by adjusting protein interaction.

Oral intake of collagen peptide has been reported to improve bone density, osteoporosis, and knee osteoarthritis [51–53]. Pro-Hyp which possesses physiological functions, is present in the blood in high concentrations after oral ingestion of collagen hydrolysate [41]. Orally administered Pro-Hyp, which is the main component of collagen peptide, is reportedly distributed in osteoblasts [10]. We previously reported that Pro-Hyp induces osteoblast differentiation by promoting Runx2 mRNA expression [36,42]. Therefore, Pro-Hyp improves bone metabolism. However, it was unclear how Pro-Hyp is involved in the transcriptional regulation of Runx2 mRNA expression. In the present study, we discovered that Pro-Hyp is incorporated into osteoblasts via Slc15a4. In addition, the present study revealed a mechanism wherein Pro-Hyp up-regulates the Runx2 distal P1 promoter via an adjusted interaction of Foxg1 and Foxo1 in mouse-derived osteoblasts. Therefore, we considered that Pro-Hyp acts as a coupling factor that modulates bone metabolism. Further studies are needed to investigate whether the same effect can be observed in human-derived osteoblasts. However, the Pro-Hyp response element of the *Runx2* distal P1 promoter that was revealed is conserved in humans and mice. Therefore, Pro-Hyp is expected to induce Runx2 mRNA expression in humans as well.

## Conclusion

**Figure 7:**
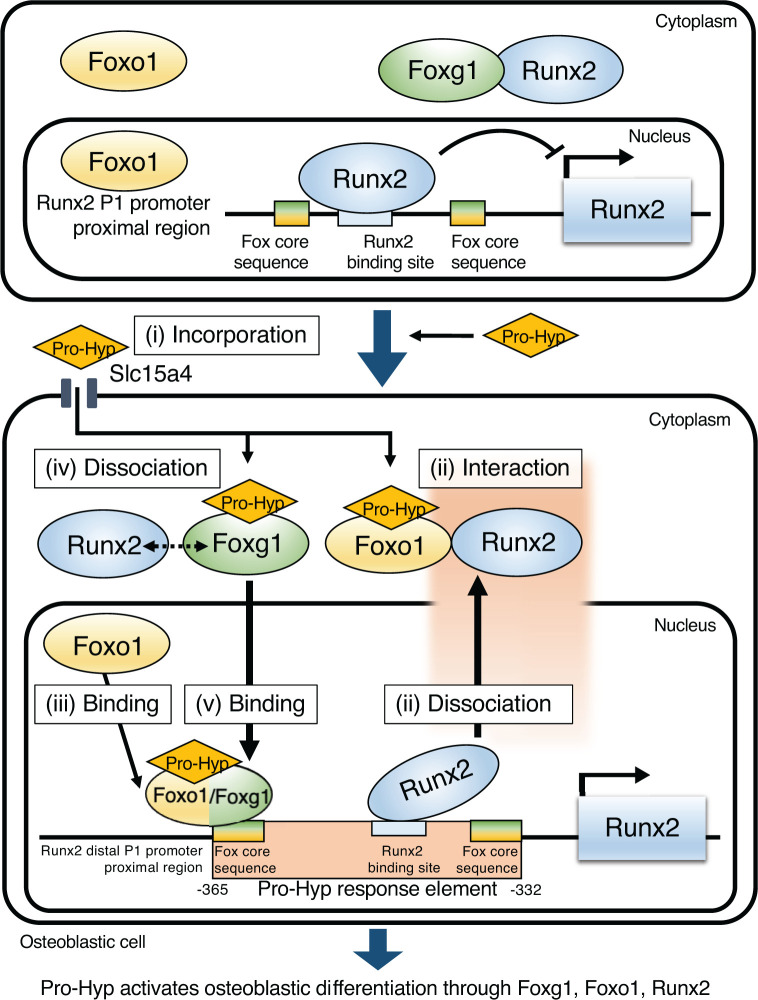
The molecular model of Pro-Hyp regulates Runx2 expression Schematic illustration of the mechanism of Pro-Hyp promoting *Runx2* P1 promoter activity through Foxg1 and Foxo1.

The present study has delineated the mechanism underlyingthe role of Pro-Hyp in osteoblast differentiation (Figure 7). In osteoblastic cells, in the absence of collagen-derived dipeptide Pro-Hyp, Runx2 binds to the proximal region of the *Runx2* distal P1 promoter, inhibiting *Runx2* expression. First, Pro-Hyp is incorporated into osteoblastic cells through Slc15a4 (Figure 7(i)). Then, Pro-Hyp binds to Foxo1, leading to their interaction with Runx2, causing Runx2 to dissociate from the *Runx2* promoter and resulting in the localization of Runx2–Foxo1 heterodimer in the cytoplasm (Figure 7(ii)). Foxo1, which has been localized to the nucleus, binds to the *Runx2* P1 promoter (Figure 7(iii)). Next, Pro-Hyp binds to Foxg1, disrupting the interaction between Foxg1 and Runx2 (Figure 7(iv)). Lastly, the dissociated Foxg1 translocates to the nucleus from the cytoplasm, binding to the *Runx2* P1 promoter and activating it (Figure 7(v)).

## Supplementary Material

Supplementary Data S1-S4

## Data Availability

All available data for the present study are contained within the manuscript. There are no external sources of data for this manuscript.

## Abbreviations

αMEM: minimum essential Eagle’s medium-α modification
ChIP: chromatin immunoprecipitation
ChIP/qPCR: chromatin immunoprecipitation/quantitative polymerase chain reaction
FBS: fetal bovine serum
FITC: fluorescein isothiocyanate
Foxg1: Forkhead box g1
Foxo1: Forkhead box o1
GST: glutathione S-transferase
IP: immunoprecipitation
MMP: matrix metalloproteinase
MT: mutation
NMTS: nuclear matrix targeting signal
PI: propidium iodide
Pro-Hyp: prolyl-hydroxyproline
Runx2: Runt-related transcription factor 2
Slc15a: solute carrier family 15 member
TLC: thin-layer chromatography
