# Supplementary material for: Stimulation of the Runx2 P1 promoter by collagen-derived dipeptide prolyl-hydroxyproline bound to Foxg1 and Foxo1 in osteoblasts
Source: Biosci Rep. 2021 Dec 7;41(12):BSR20210304. doi: 10.1042/BSR20210304 (PMC8655505; doi:10.1042/BSR20210304)
Supplement: Supplementary Data S1-S4 [file BSR-2021-0304_supp.pdf]

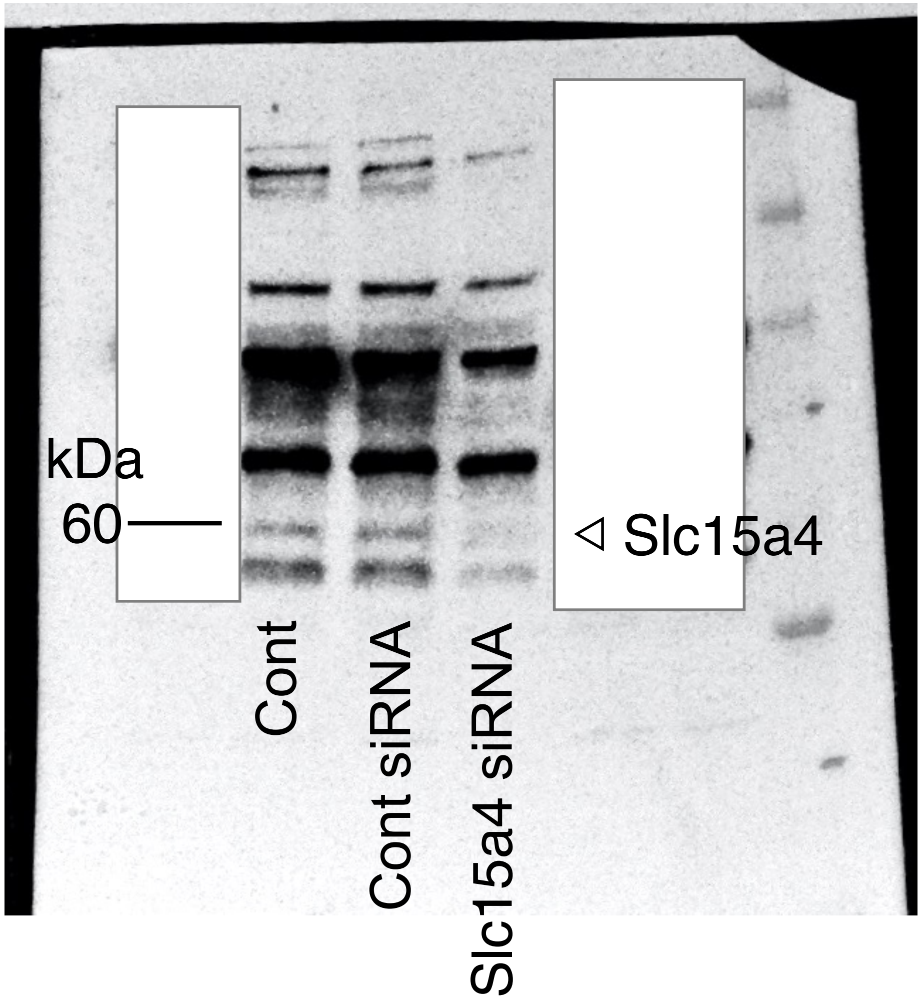

WB : Slc15a4

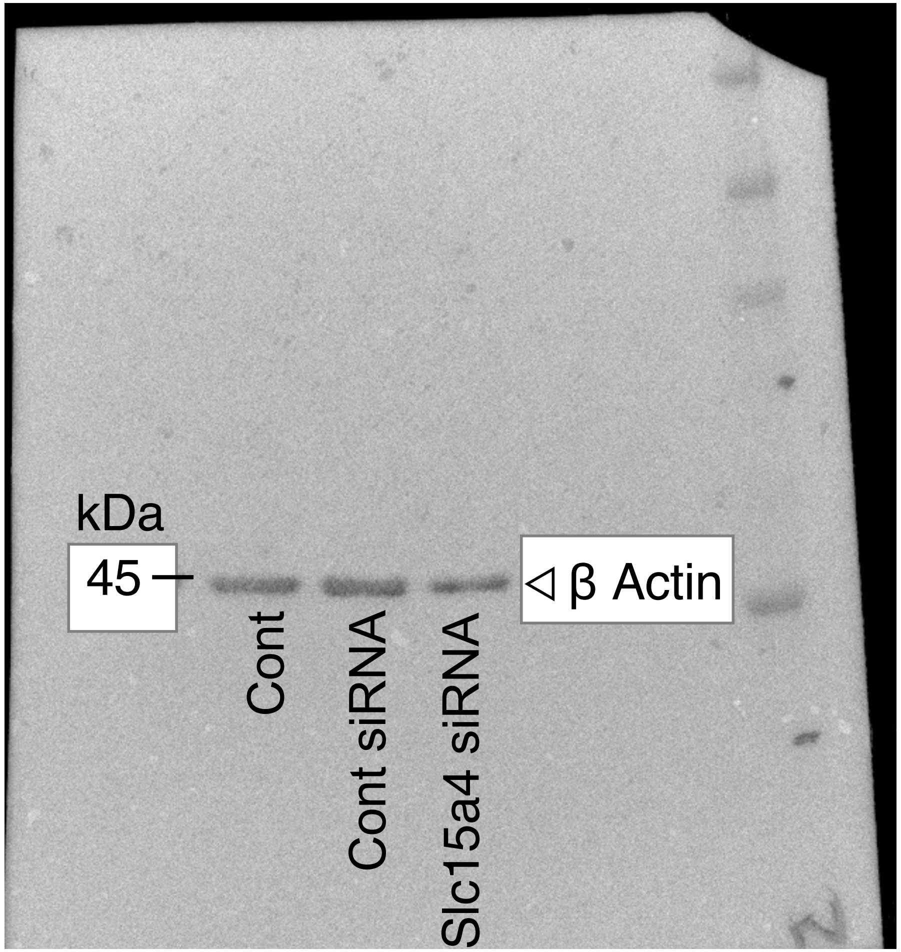

WB : β Actin

Figure 1C

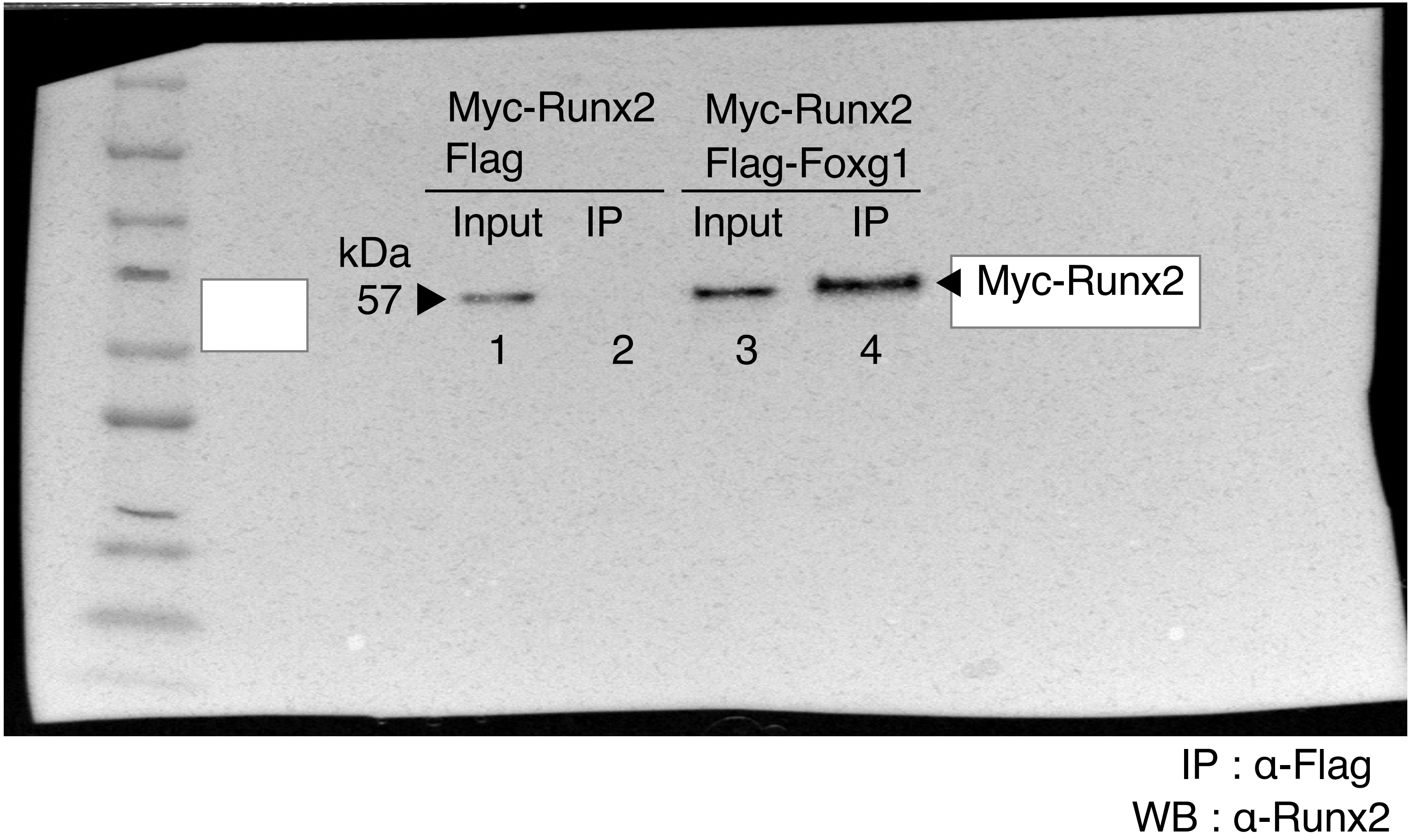
Myc-Runx2
Flag
Myc-Runx2
Flag-Foxg1
Input
IP
Input
IP
kDa
57
Myc-Runx2
1
2
3
4
IP : α-Flag
WB : α-Runx2

Figure 3A

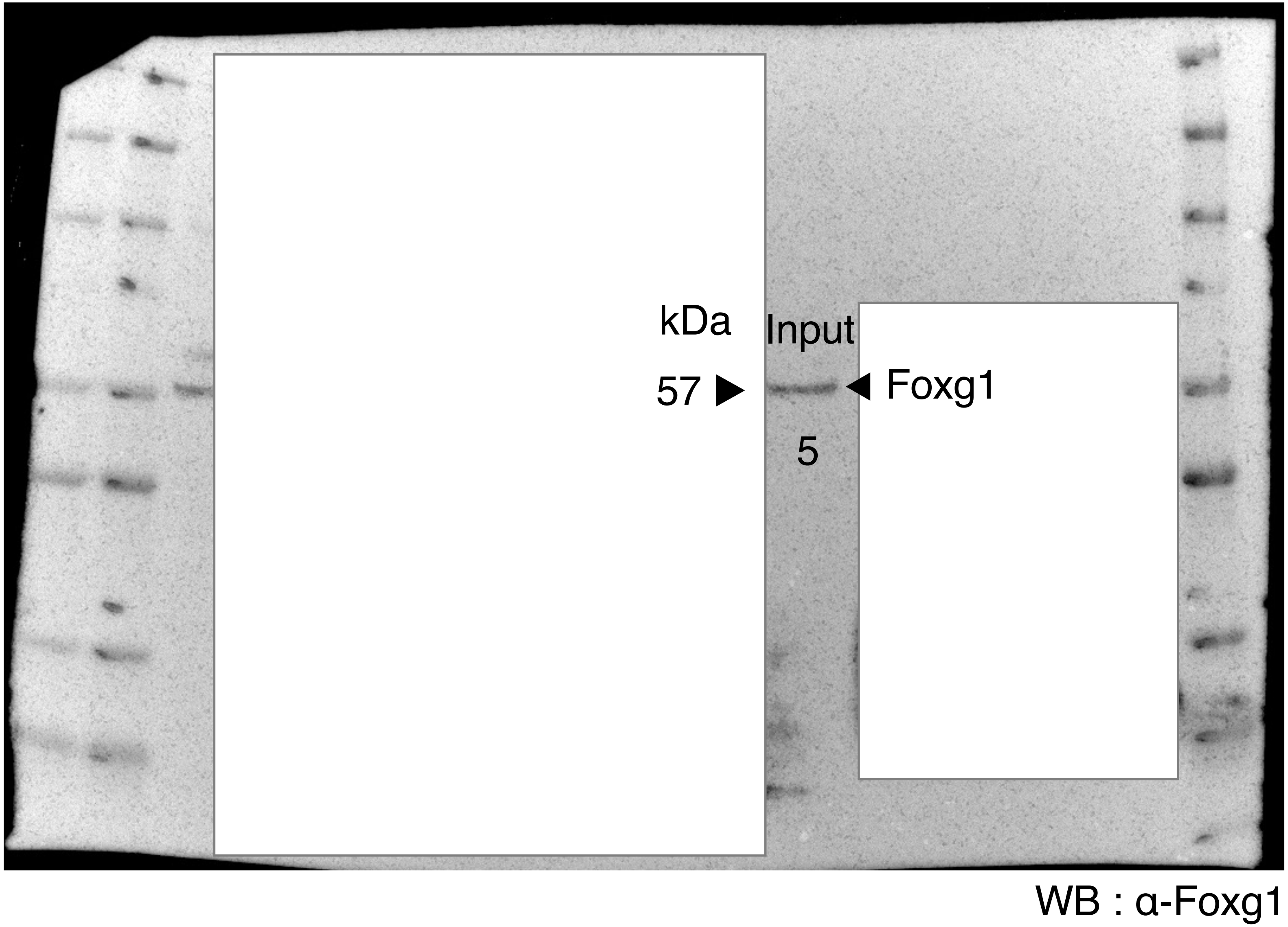

Figure 3B

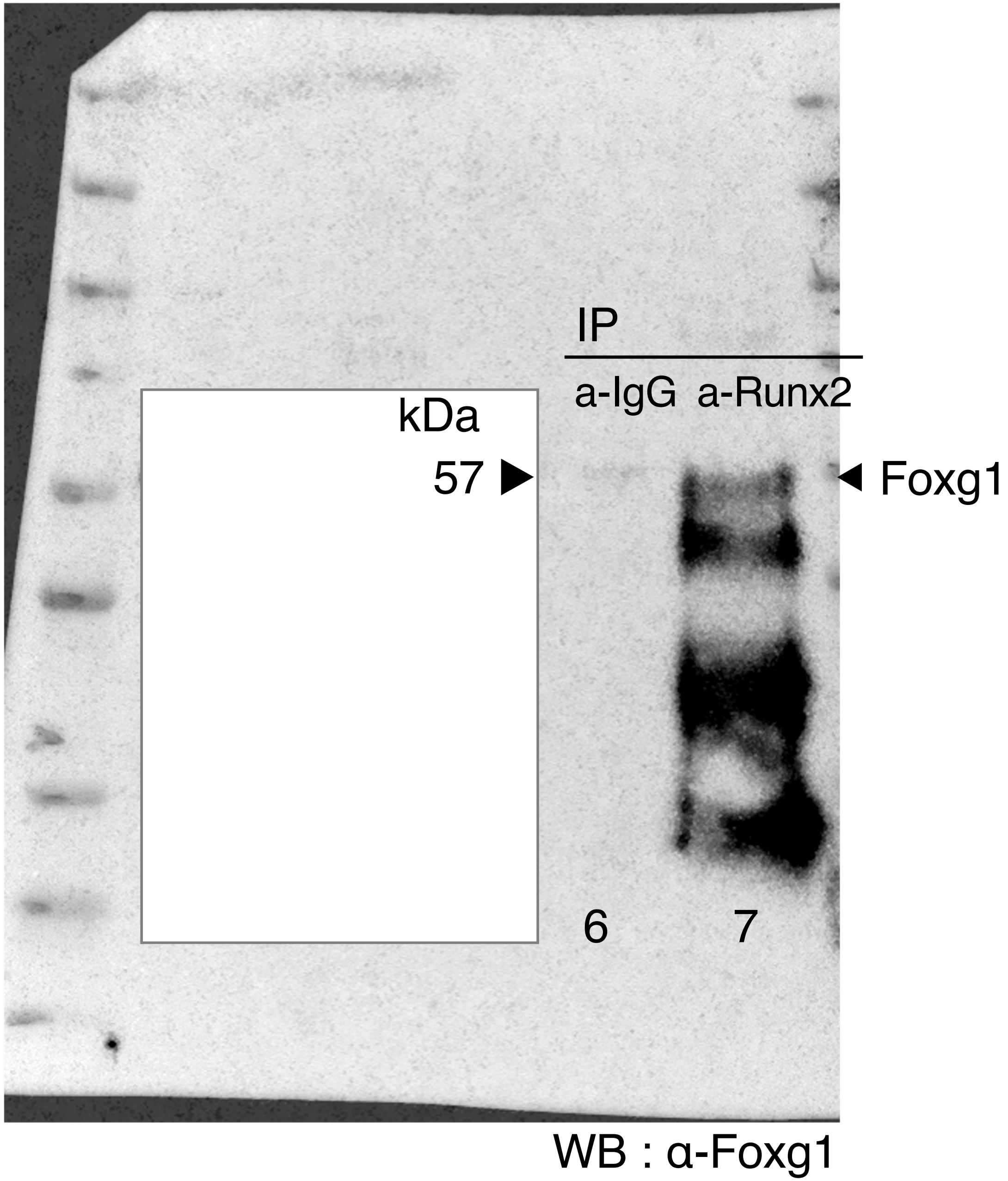

Figure 3B

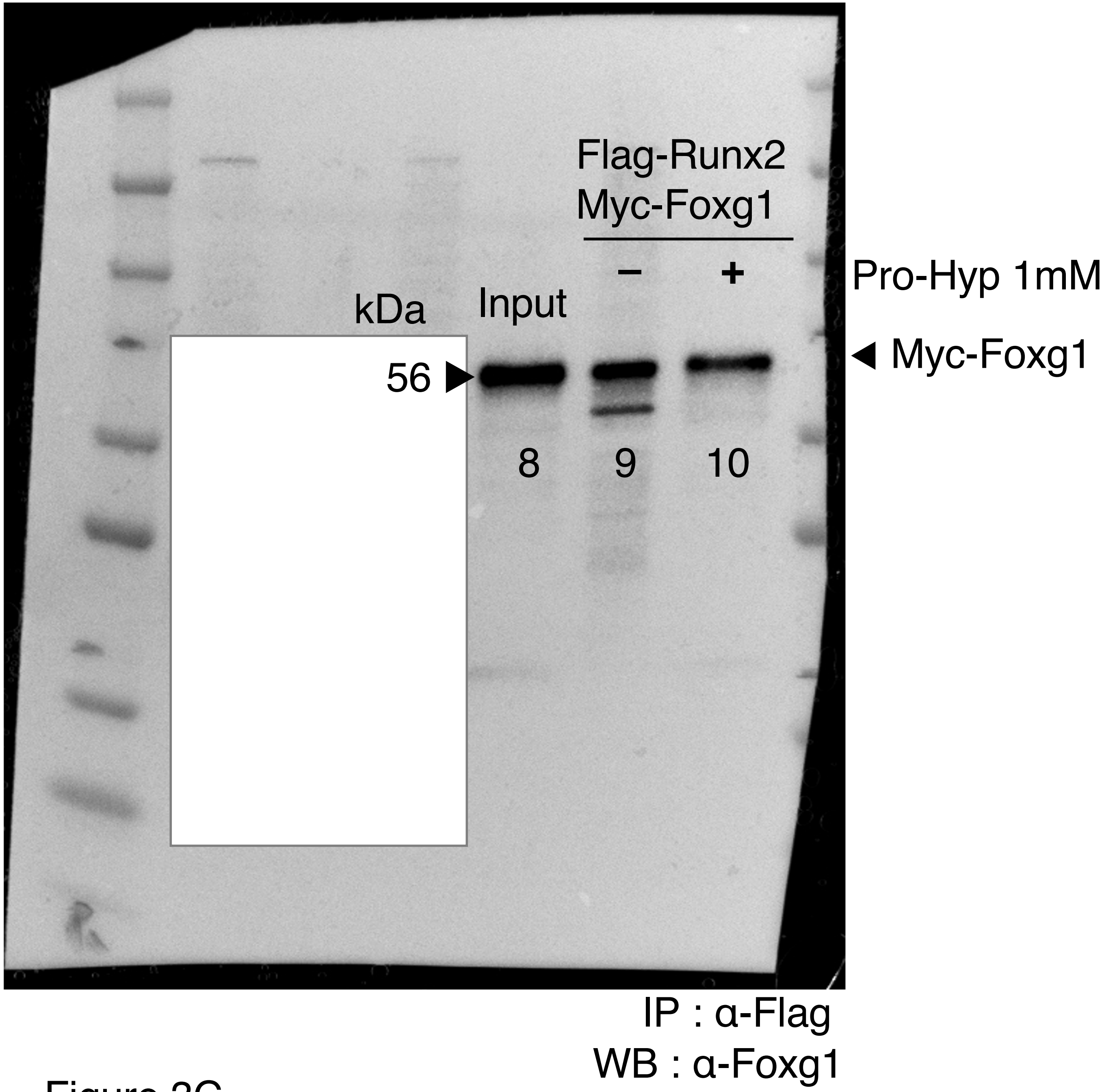
Flag-Runx2
Myc-Foxg1
–
+
Pro-Hyp 1mM
kDa
Input
56
Myc-Foxg1
8
9
10
IP : α-Flag
WB : α-Foxg1

Figure 3C

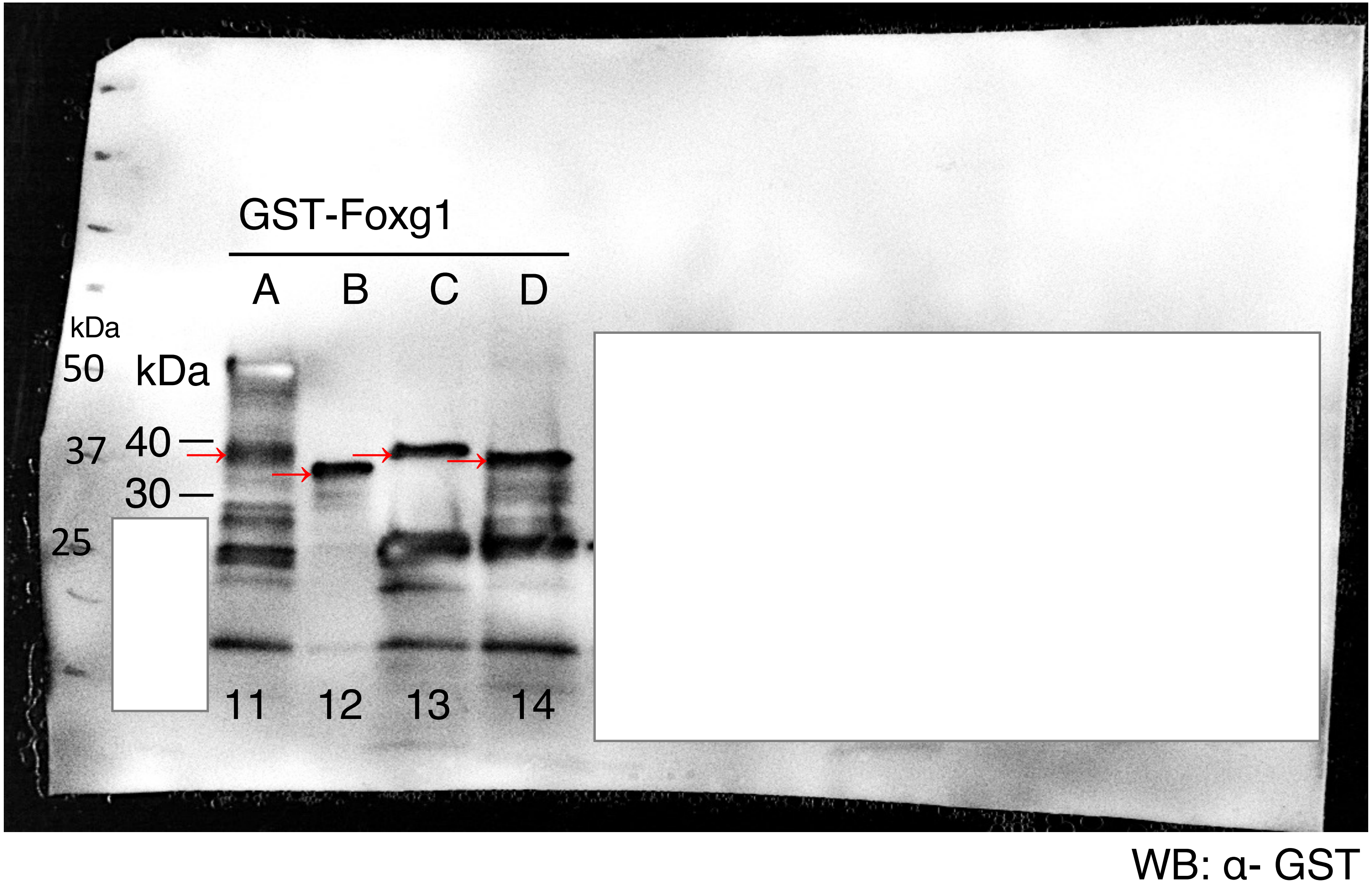

Figure 3F

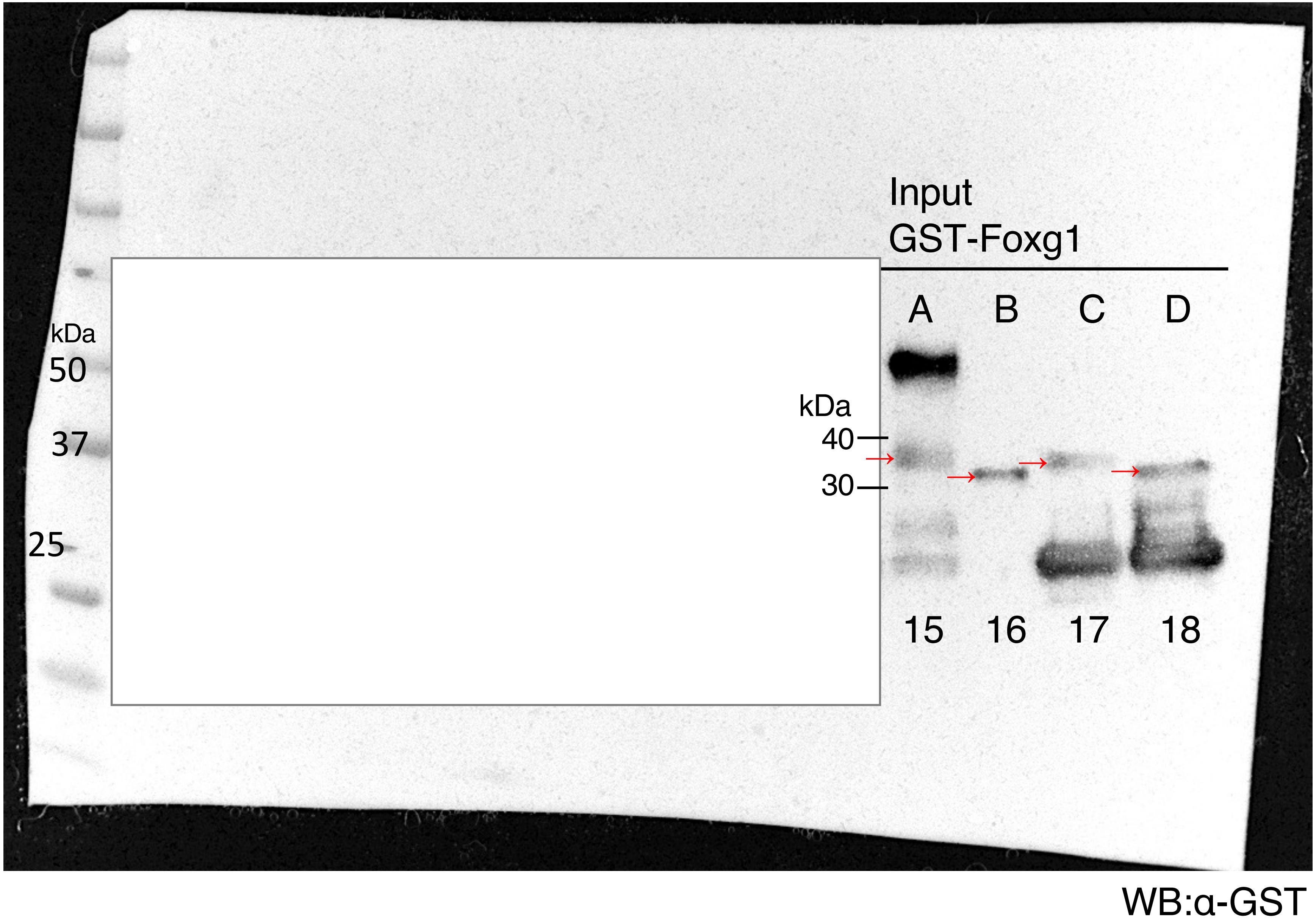

Figure 3G

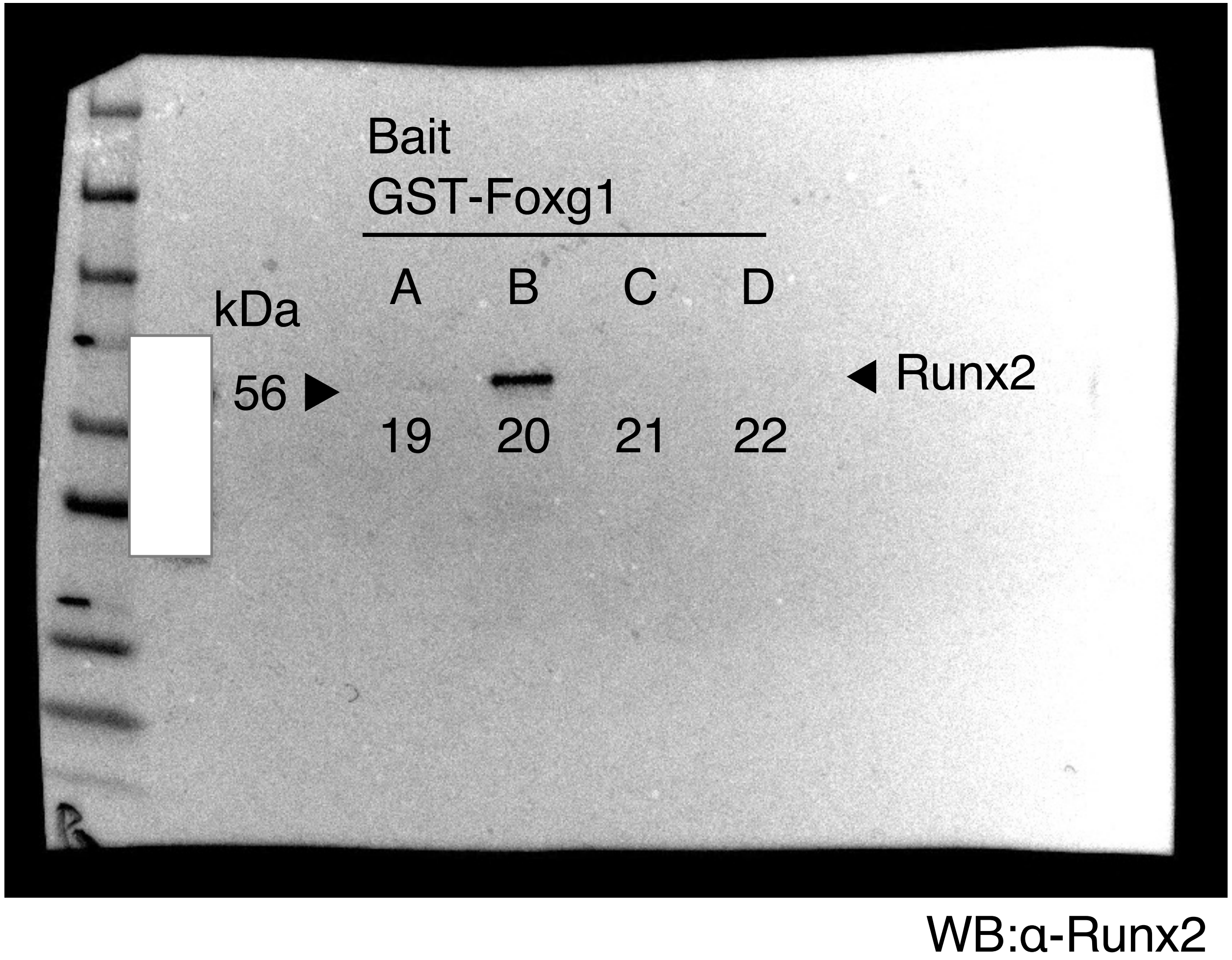

Figure 3H

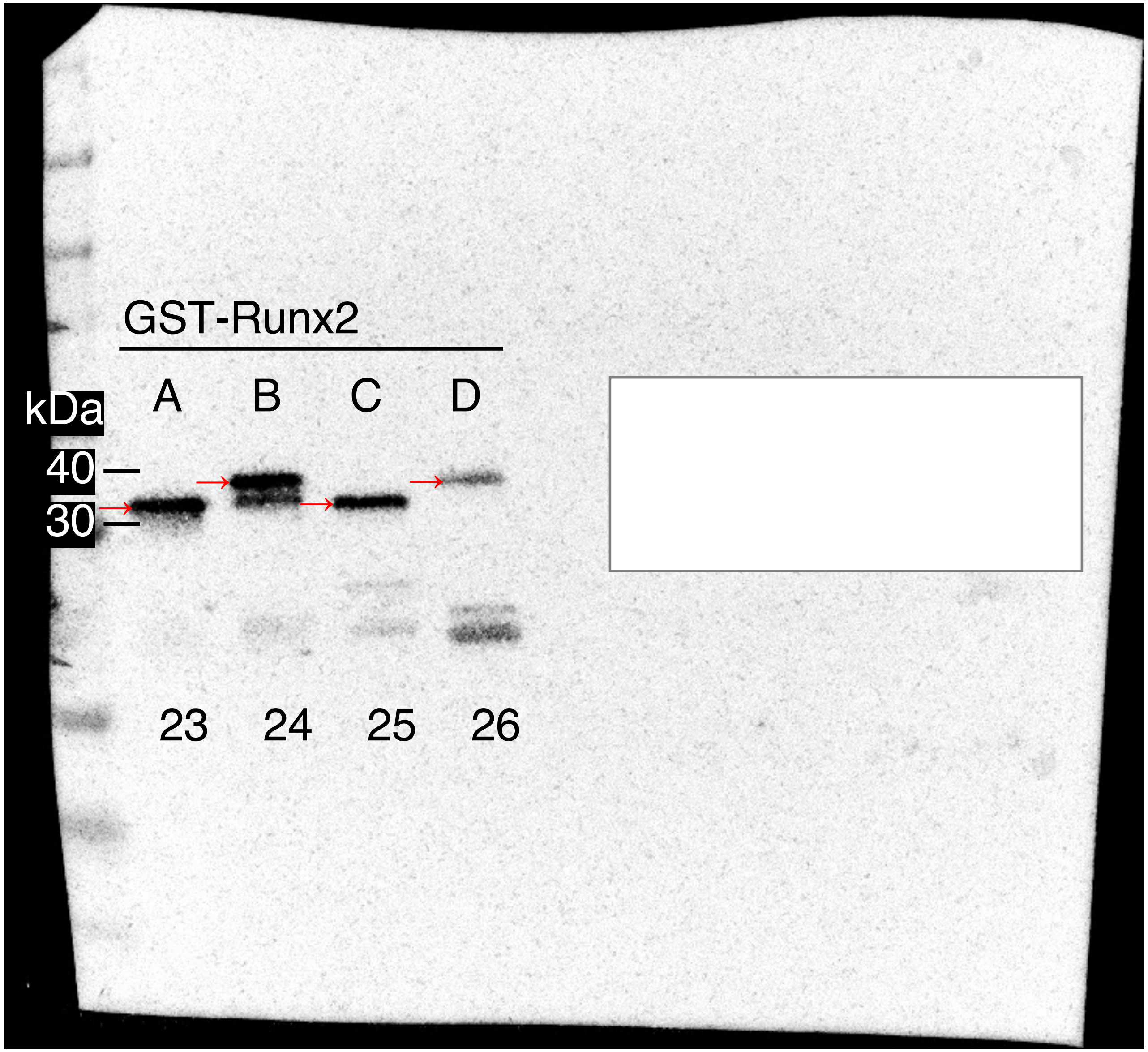

WB: α- GST

Figure 3J

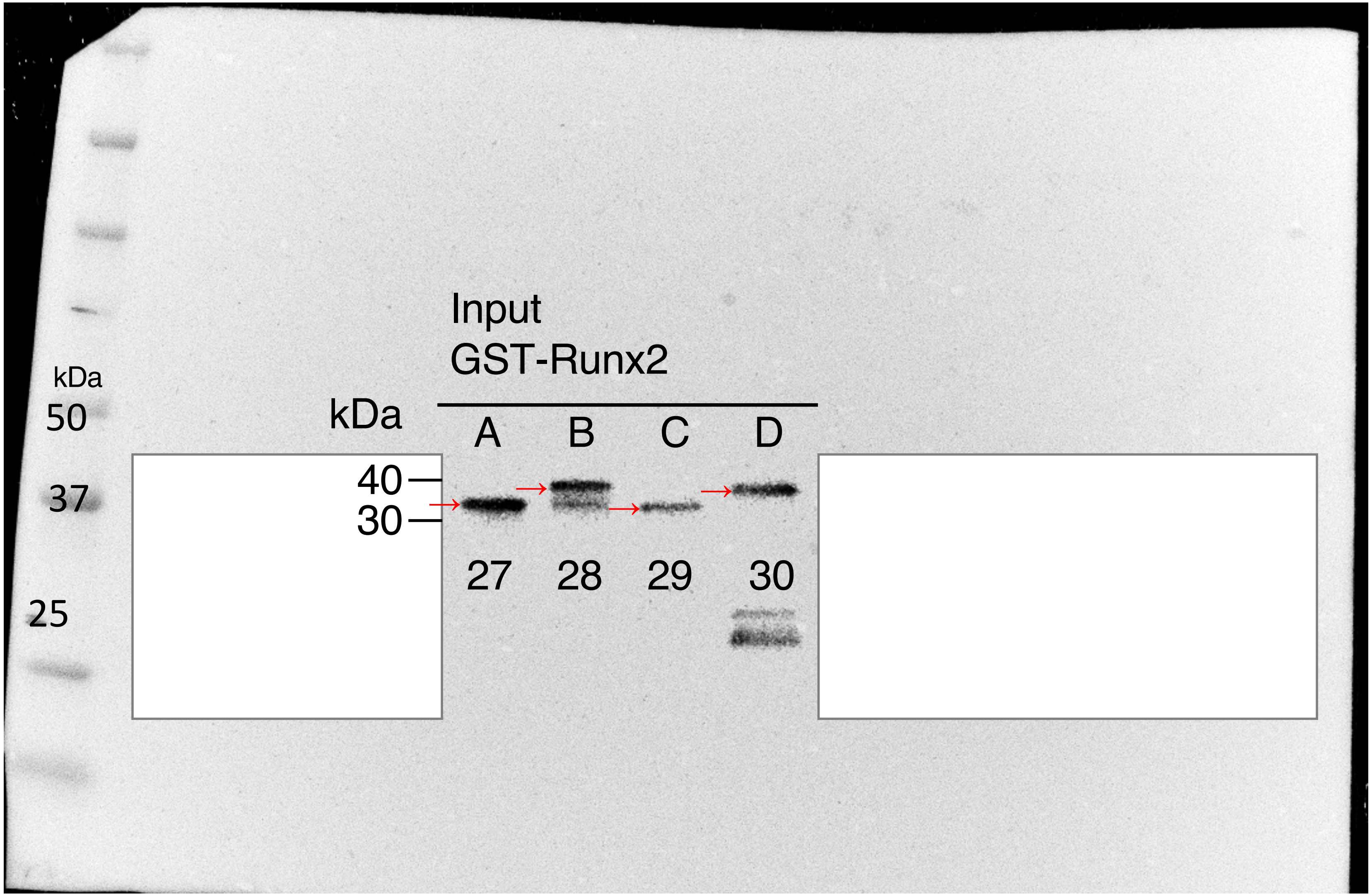

WB:α-GST

Figure 3K

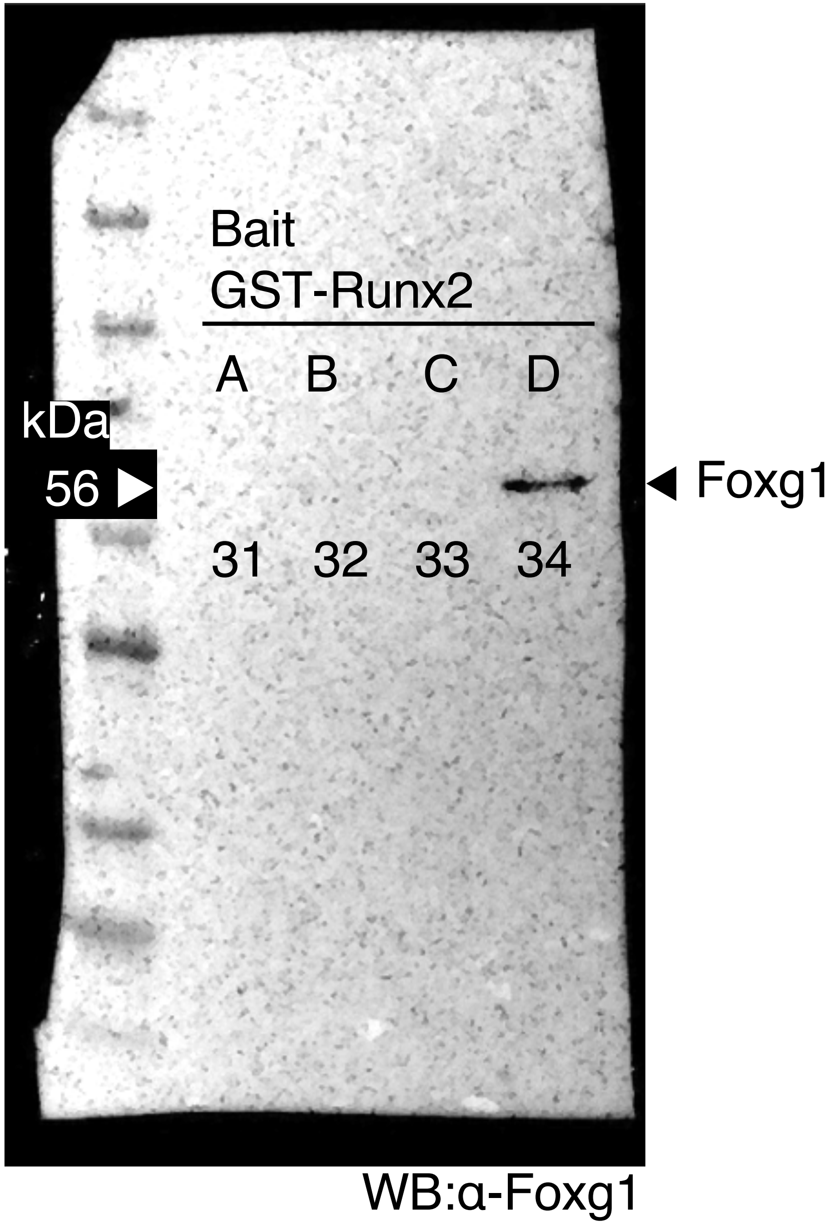

Figure 3L

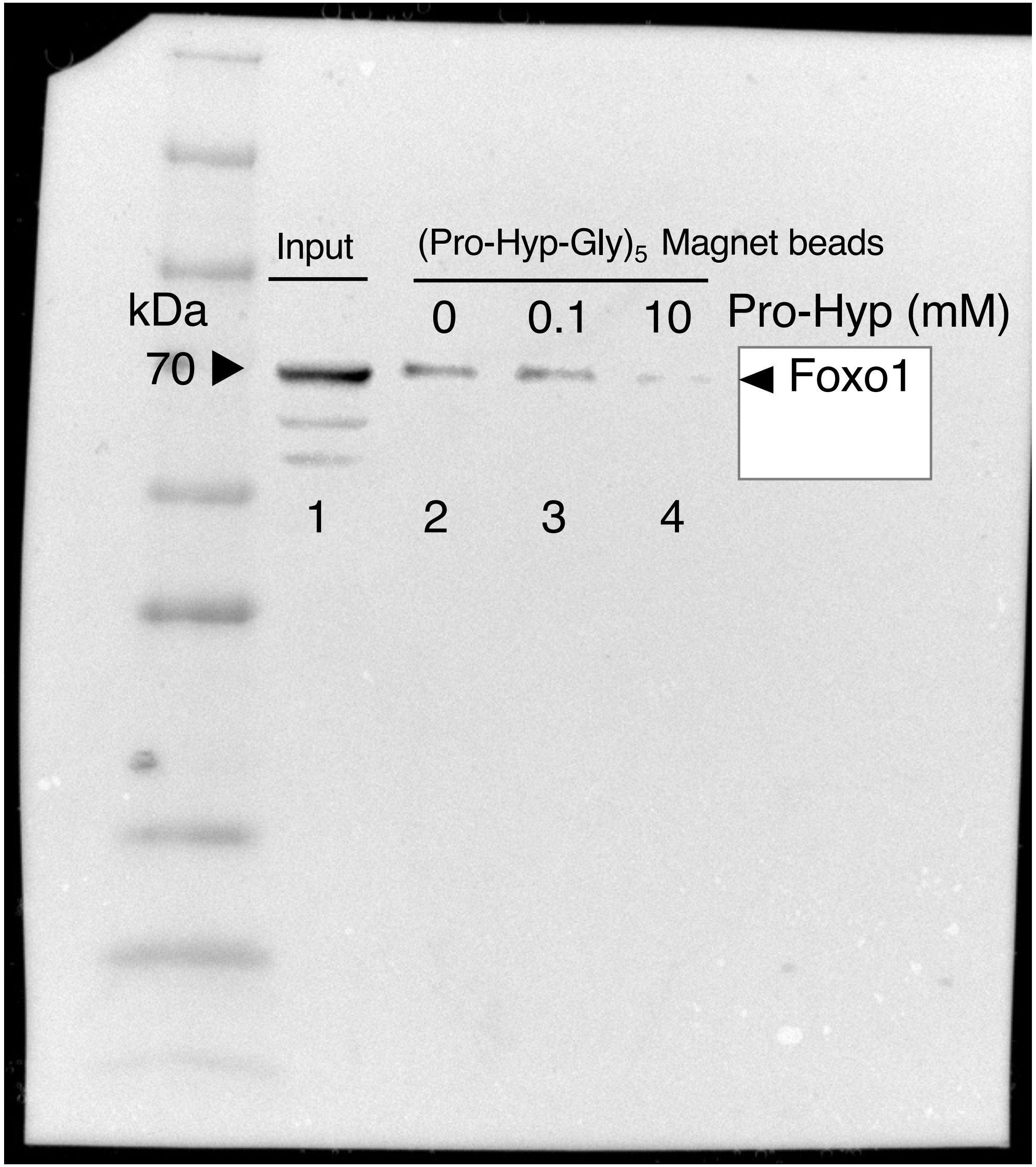

Figure 4A

WB : α-Foxo1

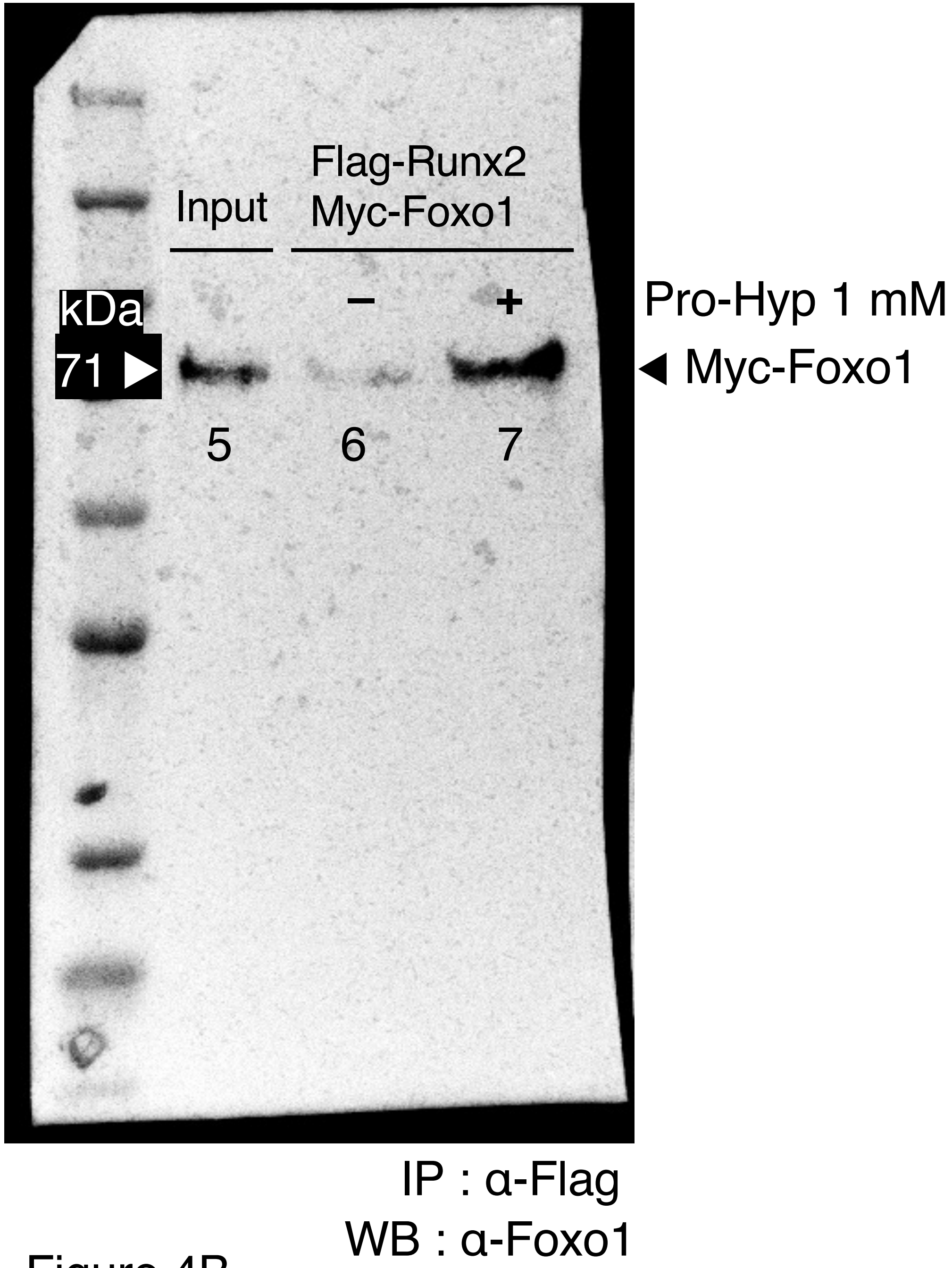
kDa
71
Input
Flag-Runx2
Myc-Foxo1
−
+
Pro-Hyp 1 mM
Myc-Foxo1
5
6
7
IP : α-Flag
WB : α-Foxo1

Figure 4B

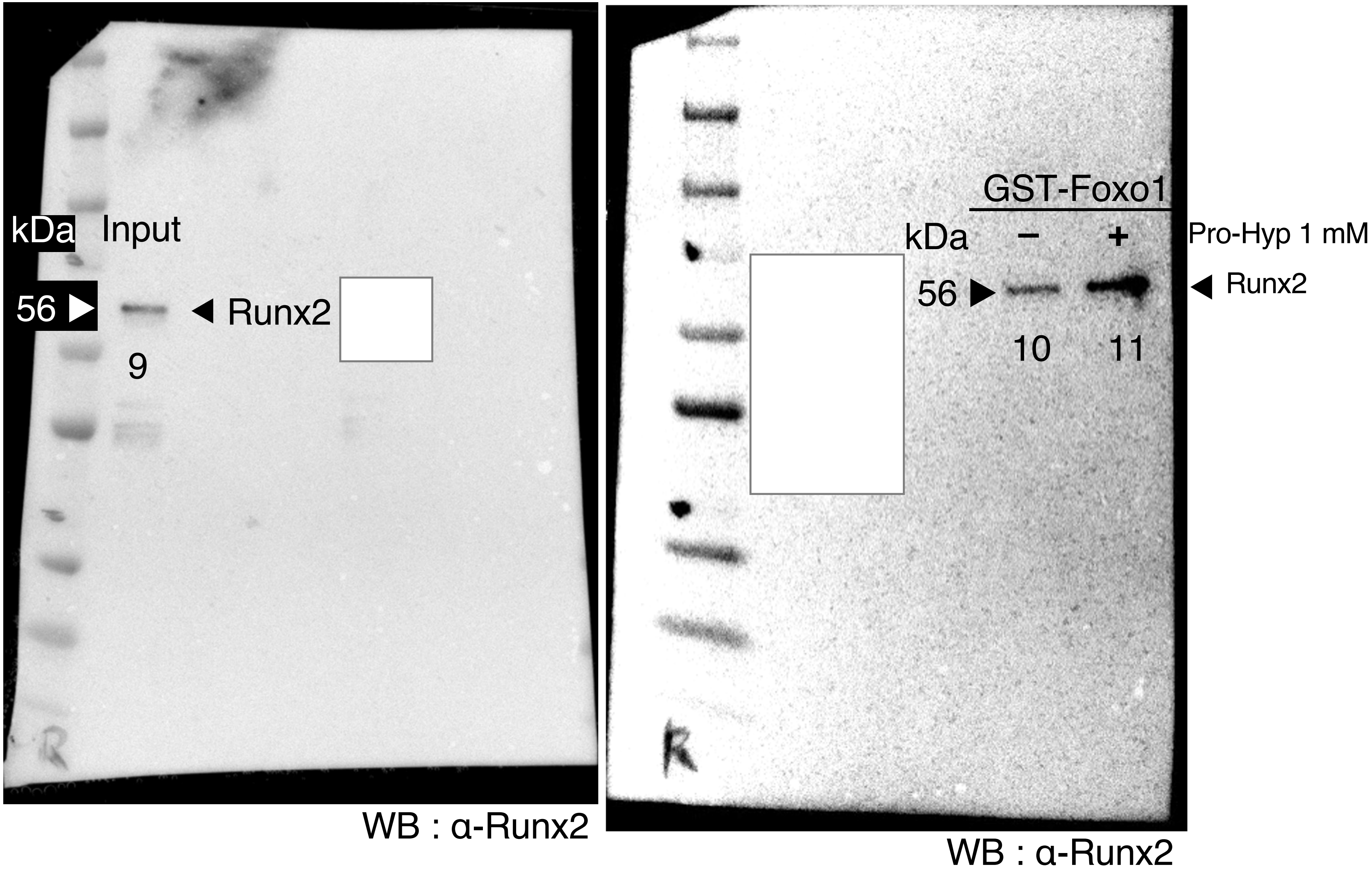
kDa
Input
56
Runx2
9
WB : α-Runx2
GST-Foxo1
kDa
−
+
Pro-Hyp 1 mM
56
Runx2
10
11
WB : α-Runx2

Figure 4D
